# Multicomponent synthesis of pyrido[2,3-*b*]pyrazine derivatives: electrochemical DNA sensing, nonlinear optical properties and biological activity†

**DOI:** 10.1039/d3ra05365b

**Published:** 2023-11-01

**Authors:** Muhammad Rashid, Muhammad Khalid, Abida Ashraf, Tahira Saleem, Iqra Shafiq, Muhammad Azeem Shakil, Briha Zainab, Attalla F. El-kott, Muhammad Yaqub, Zahid Shafiq

**Affiliations:** a Institute of Chemical Sciences, Bahauddin Zakariya University Multan 60800 Pakistan mayaqub2@yahoo.com zahidshafiq@bzu.edu.pk +92-3006559811; b Institute of Chemistry, Khwaja Fareed University of Engineering & Information Technology Rahim Yar Khan 64200 Pakistan muhammad.khalid@kfueit.edu.pk; c Centre for Theoretical and Computational Research, Khwaja Fareed University of Engineering & Information Technology Rahim Yar Khan 64200 Pakistan; d Department of Chemistry, Govt. Graduate College Shah Rukne-Alam Multan Pakistan; e Department of Biology, College of Science, King Khalid University Abha Saudi Arabia; f Department of Zoology, College of Science, Damanhour University Egypt

## Abstract

We synthesized novel pyrido[2,3-*b*]pyrazin based heterocyclic compounds (4–7) and their chemical structures were ascertained by spectral techniques (NMR, FT-IR). Besides experimental investigation, density functional theory (DFT) computations with B3LYP/6-31G(d,p) level of theory were executed to obtain spectroscopic and electronic properties. Nonlinear optical (NLO) properties, frontier molecular orbitals (FMOs), UV-visible, vibrational analysis, natural bond orbitals (NBOs), transition density matrix (TDM) and density of states (DOS) analyses of molecules (4–7) were accomplished at B3LYP/6-31G (d,p) level. Global reactivity parameters (GRPs) were correlated with the band gap (*E*_gap_) values; compound 7 with lower *E*_gap_ (3.444 eV), exhibited smaller value of hardness (1.722 eV) with greater softness value (0.290 eV^−1^). The dipole moment (*μ*), average polarizability 〈*α*〉, first (*β*_tot_) and second 〈*γ*〉 hyper-polarizabilities were calculated for compounds (4–7). Compound 7 showed less *E*_gap_, highest absorption wavelength and remarkable NLO response. The highest 〈*α*〉, *β*_tot_ and 〈*γ*〉 values for compound 7 were observed as 3.90 × 10^−23^, 15.6 × 10^−30^ and 6.63 × 10^−35^ esu, respectively. High NLO response revealed that pyrido[2,3-*b*]pyrazin based heterocyclic compounds had very remarkable contributions towards NLO technological applications. Further compounds (4–7) are utilized for the first time in electrochemical sensing of DNA, i*n vitro* antioxidant and antiurease activity.

## Abstract 2

We synthesized novel pyrido[2,3-*b*]pyrazin based heterocyclic compounds (4–7) and their chemical structures were ascertained by spectral techniques (NMR, FT-IR).

## Graphical abstract

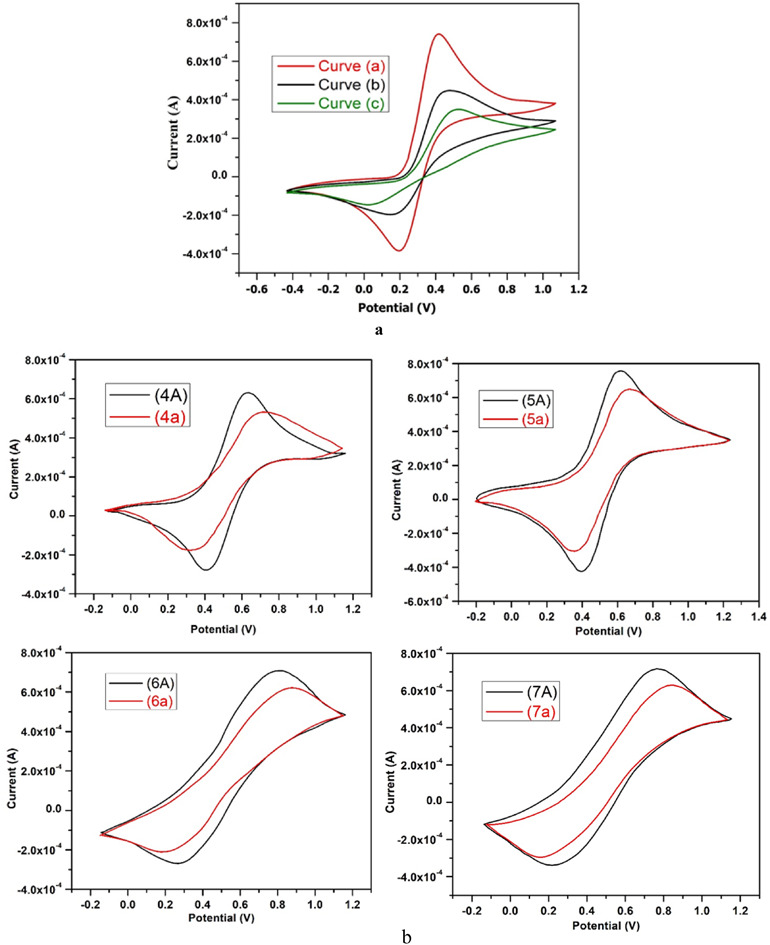

## 1. Introduction

A type of sensor uses DNA as a recognition element to detect specific molecules or analytes in a sample is called DNA electrochemical biosensors.^1,2^ The sensor is typically made of a conductive material that is coated with a layer of DNA or other biomolecules.^3^ When the DNA in the sample encounters the sensor, it can bind to the DNA on the sensor surface, causing a change in the electrical properties of the sensor. This change can be measured and used to determine the presence or concentration of the DNA in the sample. Several types of DNA electrochemical biosensors exist, including hybridization-based sensors, DNA-enzyme sensors, and DNA-modified electrode sensors.

A label-free electrochemical DNA biosensor uses an electrochemical sensor to detect and measure the presence of DNA in a sample without the need for a detectable label.^4^ It offers several advantages over traditional DNA biosensors, including high sensitivity, specificity, and the ability to detect small amounts of DNA. They are also relatively simple and inexpensive to manufacture and operate, making them an attractive option for many applications.^5^ One of the most promising applications of DNA electrochemical biosensors is in the field of medical diagnosis.^6^ These sensors have the potential to quickly and accurately detect the presence of disease-associated biomarkers,^7^ such as proteins or nucleic acids, in body fluids. This could potentially allow for the early detection of diseases, such as cancer,^8^ infectious diseases, and genetic disorders, leading to more timely and effective treatment.^9^ In addition to their medical applications, DNA electrochemical biosensors also have potential uses in environmental monitoring for example, these sensors could be used to detect the presence of harmful chemicals or contaminants in water, soil, or air.^10^

DNA electrochemical biosensors can be used to detect and measure the presence of DNA from pathogens or other contaminants in food products. This can assist to ensure the quality and safety of the food supply.^11^ DNA electrochemical biosensors can be used in forensic science to identify and quantify DNA from crime scenes or other forensic samples.^12^ Biosecurity is another field in which DNA electrochemical biosensors can be used to detect specific microorganisms or toxins that could be used in bioterrorism or other forms of sabotage.^13^

DNA electrochemical biosensors have revolutionized the field of agriculture. This can be used to monitor crop health and assess the effectiveness of agricultural practices.^14^ One of the main challenges in designing DNA electrochemical biosensors is the stability of the DNA recognition element. Researchers are working on ways to stabilize the DNA recognition element to improve the performance and longevity of DNA electrochemical biosensors.^15^ In conclusion, DNA electrochemical biosensors offer a sensitive, specific, and cost-effective means of detecting and measuring DNA in a wide range of applications.^16–19^ Out of different techniques employed for DNA biosensing, cyclic voltammetry is frequently used to detect the presence of specific DNA sequences or to measure the concentration of DNA in a sample.^20,21^ Cyclic voltammetry (CV) is an electrochemical analysing technique that includes applying a voltage to an electrode and measuring the resulting current. CV has several advantages over other DNA detection methods, including the capacity to detect extremely low DNA concentrations, great sensitivity to small fluctuations in DNA concentration, and simultaneous detection of different DNA sequences. Furthermore, it is relatively simple and does not require expensive or specialized equipment.^22^

The heterocyclic compounds with a pyrido[2,3-*b*]pyrazine core have been shown in the scientific literature to exhibit a wide range of biological applications, including selective inhibition of PI3K isozymes and, for treating myocardial infarction.^23^ They have also been explored as dipeptidyl peptidase IV inhibitors for treatment of type 2 diabetes mellitus,^24^ inhibitors of *Mycobacterium tuberculosis* growth^25,26^ antagonists for TRPV1 in pain management,^27^ fungicides,^28^ inhibitors of the Wnt/b-catenin pathway,^29^ inhibitors of PKB,^30^ ALK inhibitors,^31^ antagonists for the human neurokinin-3 (hNK-3) receptor and GnRH antagonist, as well as inhibitors of p38aMAP kinase and BRAF in cancer treatment.^31,32^ DFT calculations have attracted significant attention to predict structural parameters of novel synthesized compounds (4–7) such as non-covalent interactions, electronic properties, chemical reactivity, and stability. These results propose that the compounds under consideration have potential NLO uses. So overall, pyrido[2,3-*b*]pyrazine based compounds have shown potentials in various fields, including display technology, pharmaceuticals, and materials science.

Keeping in view the importance of DNA electrochemical biosensing and material sciences in various fields, our research group endeavors to report derivatives of indeno[2′,1′:5,6]pyrido[2,3-*b*]pyrazin (4–7), and anticipating that these molecules may acquire electrochemical DNA sensing and non-linear properties which have not yet been reported in the literature. The interaction between DNA and synthesized compounds is the fundamental biological tool for improving the therapeutic uses of organic compounds and we for the first time, determine application of compounds (4–7) in *in vitro* antioxidant and antiurease activity.

## 2. Experimental section

### 2.1 Materials and methods

Substituted aromatic aldehydes, 1,3-Indane dione, 2-aminopyrazine, p-TSA, salmon sperm DNA (SS-DNA), and different solvents were acquired from Merck/Sigma Aldrich and used as received. The melting points of synthesized compounds with fused ring heterocyclic structures were determined using an electro-thermal Gallenkamp apparatus. Thin layer chromatography (TLC) on a Merck aluminium sheet with coated silica gel was used to monitor the progress of the synthesis. The TLC plate was visualized using locating agents such as UV light with a wavelength of 366/254 nm or iodine. The FT-IR spectrometer was used to measure infrared spectra, and the 400 MHz Brauker Avance III HD 1H-NMR spectrometer was used to record 1H-NMR spectra, the solvent used was DMSO-*d*6 and internal standard was TMS. Chemical shift and coupling constant values were measured in ppm and Hz, respectively. The redox activity of DNA was measured using cyclic voltammetry (AUT50296, Autolab Potentiostat) in an electrochemical biosensor with a three-electrode system in a glass cell of 100 mL having a platinum wire, an Ag/AgCl reference electrode, and a glassy carbon electrode (GCE) with some modification along with ss-DNA as the working electrode. The working electrode passed through a cleaning process involving sonication, soap washing, triple rinsing with distilled water, and drying in an oven. A paste was prepared by blending our synthesized heterocyclic compound and ss-DNA and the in a 1 : 1 ratio (w/w) in 2 mL of ethanol. Solutions of varying concentrations were prepared in PBS (pH 7), and all the readings were taken at room temperature.

### 2.2 Computational procedure

To investigate the electronic properties, absorption spectra, and NLO characteristics of the heterocyclic compounds, we conducted DFT simulations. In a recent study, we performed quantum chemical analyses of compounds 4 to 7 at the B3LYP/6-31G(d,p) level using Gaussian 09 software packages.^33^ We employed the same level and basis set in conjunction with the Gaussian 09 embedded NBO 3.1 program package to examine natural bond orbitals (NBO) and natural populations (NPA). Similarly, we utilized time-dependent density functional theory (TD-DFT) at the B3LYP/6-31G(d,p) level for the frontier molecular orbital (FMO) analysis. GaussView 5.0 was employed for organizing input files.^34^ Additionally, Gauss View 5.0, Avogadro^35^ and Chemcraft^36^ were utilized for the elucidation of outcomes. Dipole moment was calculated by eqn (1).^37^

1 *μ* = (*μ*_*x*_^2^ + *μ*_*y*_^2^ + *μ*_*z*_^2^)^1/2^

Average polarizability was calculated with eqn (2).^38^

2 〈*α*〉 = (*a*_*xx*_ + *a*_*yy*_ + *a*_*zz*_)/3

Gaussian outcome analysis file contains ten hyper-polarizability tensors oriented in the *x*, y, and *z* directions: *β*_*xxx*_, *β*_*xyy*_, *β*_*xzz*_, *β*_*yyy*_, *β*_*xxy*_, *β*_*yzz*_, *β*_*zzz*_, *β*_*xxz*_, *β*_*yyz*_, *β*_*xyz*_. First hyper-polarizability (*β*_tot_) is calculated with the help of eqn (3).^39^

3 *β*_tot_= (*β*_*x*_^2^ + *β*_*y*_^2^ + *β*_*z*_^2^)^1/2^

Where, *β*_*x*_ = *β*_*xxx*_ + *β*_*xyy*_ + *β*_*xzz*_, *β*_*y*_ = *β*_*yxx*_ + *β*_*yyy*_ + *β*_*yzz*_ and *β*_*z*_ = *β*_*zxx*_ + *β*_*zyy*_ + *β*_*zzz*_.

The second hyper-polarizability was calculated with eqn (4).^38^4
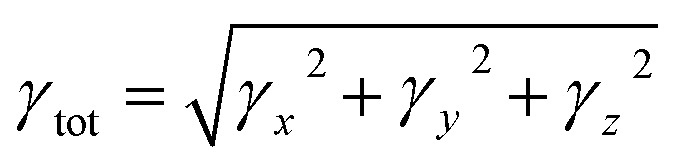


### 2.3 Electrochemical DNA sensing

#### 2.3.1 Sample preparation

Two milligrams of salmon sperm DNA were solubilized in distilled water and subjected to overnight agitation at a temperature of 4 °C. The genomic material was subsequently introduced into a buffer solution, yielding an absorbance ratio of 1.8 at wavelengths of 260 and 280 nanometers (nm). This outcome signifies the absence of any protein impurities within the DNA sample.^40^

#### 2.3.2 Cyclic voltammetry of K_3_[Fe(CN)_6_]

K_3_[Fe(CN)_6_] can act as a facilitator of electron transfer in electrochemical biosensors. We discovered more about electrochemical behaviour of the K_3_[Fe(CN)_6_] as a redox mediator^41^ by taking a cyclic voltammogram of given solution which have 2.0 mmol L^−1^ of K_3_[Fe(CN)_6_], 0.50 mol L^−1^ of KNO_3_ and, 0.01 mol L^−1^ of PBS.

### 2.4 Biological activity assay

#### 2.4.1 *In vitro* antioxidant activity

The assessment of antioxidative properties in compounds characterized by nitrogen-containing heterocyclic derivatives of pyrido[2,3-*b*]pyrazine was conducted using a previously documented experimental protocol established by Subbareddy and colleagues.^42^ Spectrophotometric analysis was used to assess the radical scavenging activity of substances against stable DPPH (2,2-diphenyl-2picrylhydrayl hydrate). It gets reduced when DPPH combines with an antioxidant molecule that can give hydrogen. Color variations (from deep violet to pale yellow) were measured using a UV-vis light spectrophotometer at 517 nm. In a chemical context, a DPPH solution is subjected to interaction with a substance possessing the capacity to donate a hydrogen atom, leading to the formation of the reduced state and concomitant disappearance of its violet hue. The reaction is:DPPH˙ + RH → DPPH + R˙

1 mg of a chemical compound was solubilized in 1 mL of ethanol to generate initial stock solutions. Subsequently, this substance was dissolved in 3 mL of ethanol, resulting in solutions with varying concentrations of 10, 50, and 100 g. Just before the UV measurements, a DPPH solution in ethanol was prepared. Following that, 3 mL of the sample and 1 mL of the DPPH solution were mixed and left in the dark for 30 minutes at room temperature before measuring the decrease in absorption. A blank sample containing the same amount of ethanol and DPPH solution was used to test the control absorption. Ascorbic acid was employed as the reference. The experiment was done three times. The scavenging activity increased as the concentration of the test samples increases.

The radical scavenging activity was calculated by the following formula:% Inhibition = [*A*_b_ − *A*_a_/*A*_b_] × 100where *A*_b_ is the absorption of the blank sample and *A*_a_ is the absorption of the sample.

#### 2.4.2 *In vitro* antiurease activity

The enzyme responsible for catalyzing urea, known as urease, helps for the conversion of urea into carbon dioxide (CO_2_) through hydrolysis. To monitor the production of ammonia (NH_3_) and inhibit urease activity, we employed the indophenol method.^42^ In the reaction mixture, consisting of Jack Bean Urease (200 μL), a buffer with a pH of 8.25 (400 μL, composed of 0.5 mM EDTA, 0.02 M K_2_HPO_4_, 0.02 M LiCl, and 120 mM urea), and test compounds (100 μL), incubation was carried out at 25 °C for approximately 30 minutes. Following the incubation, in each test tube phenol reagent (500 μL, containing 0.006% w/v sodium nitroprusside and 2% w/v phenol) and an alkali reagent (600 μL, comprising 0.15% v/v NaOCl and 0.1% w/v NaOH) were poured, and after a 40 minutes of incubation period, the results were recorded by measuring the absorbance at 625 nm using a UV-vis spectrophotometer (1601UV Shimadzu, Australia).

Percentage inhibition was determined using the following formula:% Inhibition = [Control OD − (Sample OD/Control OD)] × 100

In the realm of scientific investigation, thiourea functions as a benchmark inhibitor. The determination of the IC_50_ values entails subjecting both standard and synthesized compounds to varying concentrations under identical experimental conditions.^43,44^

### 2.5 Synthetic procedure

#### 2.5.1 General procedure for the synthesis of compounds (4–7)

**Scheme 1:**
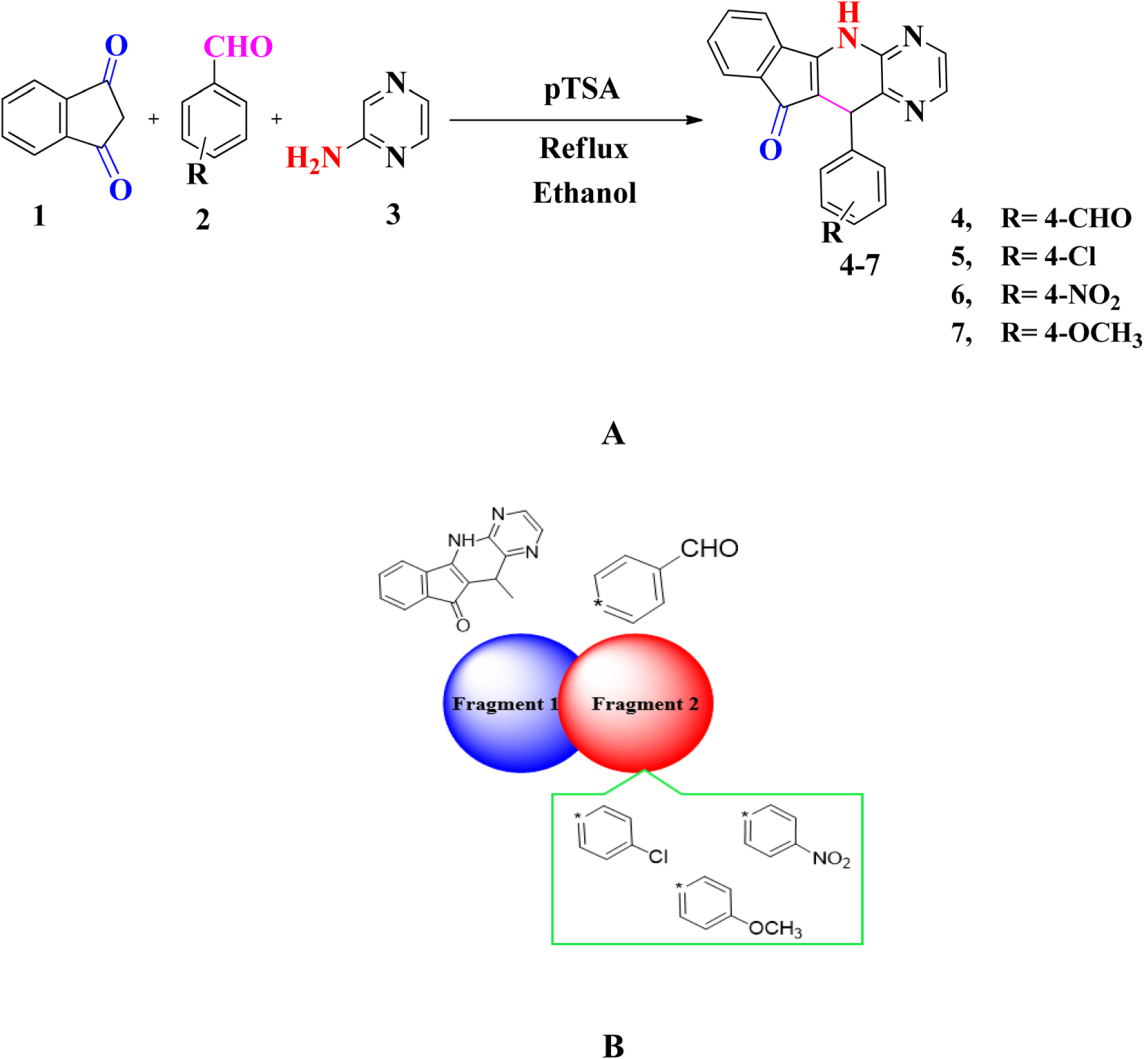
(A) Synthesis of indeno[2′,1′:5,6]pyrido[2,3-*b*]pyrazin (4–7) (B) sketch map of studied compounds (4–7).

The three components, 0.684 mmol of a substituted aromatic aldehyde, 0.1 g (0.684 mmol) of 1,3-indane dione, 0.684 mmol of 2-aminopyrazine, and a catalyst 20% *p*-TSA were placed in a round bottom flask containing a magnetic stirrer and refluxed in 10 mL of ethanol. After approximately 8 hours of reflux, the reaction was deemed complete based on TLC analysis using a solvent mixture of 70% petroleum ether and 30% ethyl acetate. The solution was subsequently permitted to undergo gradual cooling to reach ambient temperature, resulting in the formation of yellowish solid precipitates characteristic of the anticipated pyrazine derivative derived from a heterocyclic compound. These precipitates were subsequently subjected to filtration and subsequent washing with both water and low-temperature ethanol. The product was then recrystallized using ethyl acetate and dried under a vacuum overnight at room temperature. The resulting pure heterocyclic compound, referred to as compound 4–7 in yield of 82–89% (Scheme 1).

## 3. Results and discussion

### 3.1 Chemistry

A series of substituted pyrido[2,3-*b*]pyrazine based heterocyclic compounds (4–7) have been synthesized to explore the DNA electrochemical sensing, NLO properties and biological screening.

These compounds are prepared by reacting a mixture of indane 1,3 dione (1), substituted aromatic aldehyde (2) and 2-aminopyrazine (3) with 20 mol% *p*-TSA in ethanol. The optimization of reaction conditions is done for model compound 7 by treating 4-methoxybenzaldehyde, 2-aminopyrazine and indane 1,3 dione in equimolar amounts by using solvents of different polarity *i.e.* H_2_O, ethanol, DCM, THF, CH_3_CN and DMF. Furthermore, the reaction is also carried out in the presence and absence of catalyst. Thus after several attempts the reaction conditions defined to obtain pyrido[2,3-*b*]pyrazine based heterocyclic compounds (4–7) is shown in entry 8 (20 mol% *p*-TSA, 9 h) with yield of 89% (Table S1†). The substrate scope of reaction is extended vide reacting indane 1,3 dione (1), different aromatic aldehydes (2) and 2-aminopyrazine (3). The targeted compounds (4–7) are obtained in good to excellent yield (82–89%) (Table S2†) (Scheme 1A).

The structure of novel pyrido[2,3-*b*]pyrazine based heterocyclic compounds (4–7) are established by using FTIR, NMR (^1^H & ^13^C) and CHN analysis. The NH stretching in FTIR appear in the range of 3193–3459 cm^−1^ while the peaks in range of 1566–1661 cm^−1^ showed the presence of C=O group. The ^1^H NMR spectra of compounds (4–7) show the presence of singlet for (CH) non-aromatic proton in the range of 5.46–5.64 ppm while the singlet peak in the range of 8.95–9.62 ppm unveiled the presence of NH proton. Total count of proton in aromatic region also justifies the structural requirement of compounds. The ^13^C NMR spectra of compounds (4–7) supports the FTIR and ^1^H NMR data. The proposed mechanism of reaction is shown in Scheme S1a.†

### 3.2 Electrochemical DNA sensing study

#### 3.2.1 Optimization the immobilization of salmon sperm (ss-DNA)

**Fig. 1:**
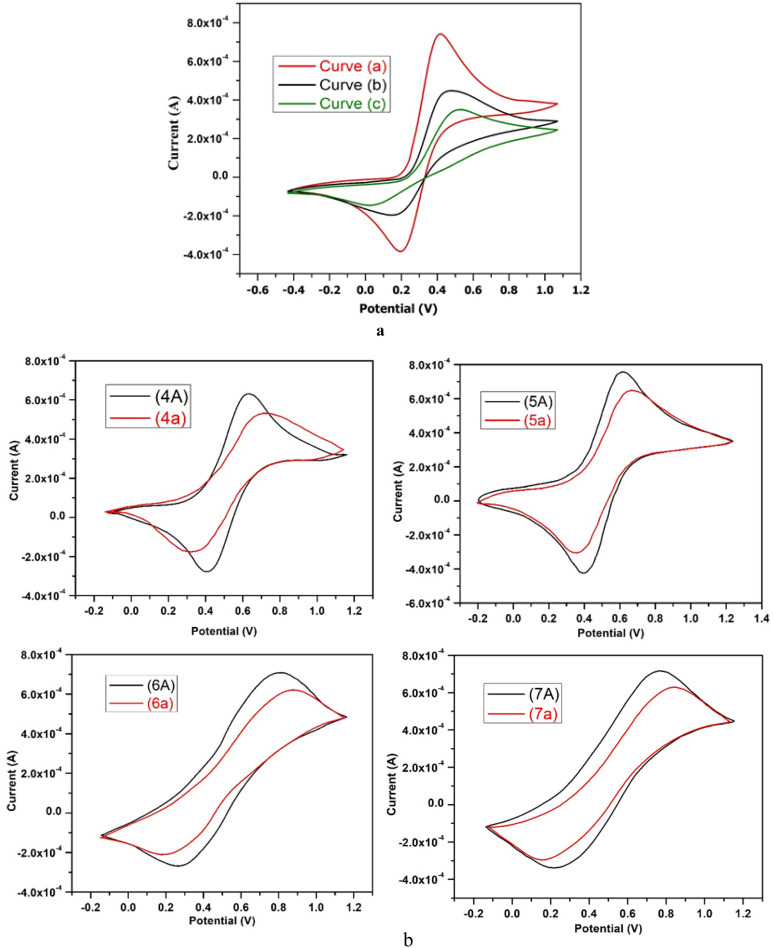
(a) CV curves of (a) glassy carbon electrode (GCE) (b) bare electrode and (c) after immobilization of ss-DNA on glassy carbon electrode (GCE) in 0.1 M PBS (pH 7), 0.1 M KCl, 0.001 M K_3_Fe(CN)_6_, and scan rate 100V s^−1^ with scanning range from −0.7 V to 1.2 V. (b) CV curves in the absence of ss-DNA (curve, 7A) and in the presence of 10 mg L^−1^ of ss-DNA (curve, 7a) in 0.1 M PBS (pH 7), 0.1 M KCl, 0.001 M K_3_Fe(CN)_6,_ and scan rate 100V s^−1^, scanning range from −0.3 V to 1.3 V.

The cyclic voltammetry (CV) technique along with the indicator Fe(CN)_6_^3−^/Fe(CN)_6_^4−^ redox couple, was employed to examine the immobilization of ss-DNA on the surface of a glassy carbon electrode (GCE). The said indicator works on the basis of repulsive interaction between the negatively charged DNA phosphate backbone and the redox couple.^45^ As shown in Fig. 1a, the immobilization process is represented by typical cyclic voltammograms. In the case of using a bare electrode, the voltammogram of K_3_[Fe(CN)_6_] displays two clear peaks, a cathodic peak with potential (*E*_pc_) of 150 mV and an anodic peak with potential (*E*_pa_) of 490 mV. The separation between these peak-to-peak potentials (Δ*E*_p_) is 340 mV, represented by the curve “b” in Fig. 1a. The current of both peaks increased and the reversibility of the redox reaction of the Fe(CN)_6_^3−^ redox process was enhanced when using a glassy carbon electrode (GCE). Furthermore, the separation between these peak-to-peak potentials (Δ*E*_p_) decreased to 190 mV, as shown by curve “a” in Fig. 1a.

When ss-DNA is immobilized on the surface of a glassy carbon electrode (GCE), it causes a decrease in the electrochemical reversibility of the Fe(CN)_6_^3−^/Fe (CN)_6_^4−^ redox couple, and an increase is shown by Δ*E*_p_ to 470 mV (curve “c” in Fig. 1a). Further, an increase in charge transfer resistance and a decrease in the peak current of the redox probe [Fe(CN)_6_]^3−^/^4−^ occur as a result of contact between the amine groups of ss-DNA bases and the carboxylic groups of the modified GCE surface.^46^ Additionally, the immobilization of ss-DNA on the GCE surface results in an enhancement of the specific surface area and electrical conductivity of the sensing platform, leading to a lower peak potential for the electrooxidation of guanine residues in ssDNA.^47^ Due to electrostatic repulsion, the phosphate groups which are negatively charged on the ss-DNA may inhibit the Fe(CN)_6_^3−^/Fe (CN)_6_^4−^ redox pair from accessing the electrode surface. The difference in CV profiles between both electrodes can be exploited in this investigation to detect ss-DNA immobilisation on the electrode surface.^41^

#### 3.2.2 Interaction between synthesized compounds and the salmon sperm, ss-DNA in solution

While discussing the cyclic voltammetry data of our synthesised compound 4, we detected two distinct anodic and cathodic peaks with peak potentials of 620 mV and 400 mV, respectively, in the absence of ss-DNA. The separation between these peak-to-peak potentials (*E*_p_) was 220 mV, as shown in Fig. 1b by the black coloured curves 4A. With the addition of ss-DNA, the current of both peaks reduced. Anodic and cathodic peak potentials both were increased to higher positive and negative values, respectively, as was the peak-to-peak potential. The anodic and cathodic peaks potentials are now 705 and 320 mV, respectively, with a 385 mV separating between these peak-to-peak potentials (*E*_p_), as illustrated by curve 4a in Fig. 1b.

We found two distinct anodic and cathodic peaks with peak potentials of 505 mV and 400 mV, respectively, when using cyclic voltammetry to analyse the results of our synthesised heterocyclic compound 5. The black coloured curves 5A in Fig. 1b depict this peak-to-peak potential separation (*E*_p_), which was 105 mV. When ss-DNA was added, the current of both peaks reduced. Peak-to-peak potential increased, and anodic and cathodic peak potentials both were improved towards more positive and more negative values, respectively. The spacing between these peak-to-peak potentials (*E*_p_) has now increased to 290 mV, as illustrated by curve 5a in Fig. 1b, and the anodic and cathodic peaks potentials are now 650 mV and 360 mV, respectively.

When discussing the cyclic voltammetry data of our synthesised compound 6, we found two distinct anodic and cathodic peaks with peak potentials of 800 mV and 290 mV, respectively, in the absence of ss-DNA. The black coloured curves 6A in Fig. 1b reflect this peak-to-peak potential separation (*E*_p_), which was 510 mV. With the addition of ss-DNA, the current of both peaks reduced. Along with an increase in peak-to-peak potential, the anodic and cathodic peak potentials were both increased towards more positive and more negative values, respectively. As indicated by curve 6a in Fig. 1b, the anodic and cathodic peaks potentials now change to 870 mV and 210 mV, respectively, with an increased peak-to-peak potential separation (*E*_p_) of 660 mV.

The effect of ss-DNA on the electrochemical behavior of (synthesized compound, 7) in 0.1 M PBS (pH 7) was examined using cyclic voltammetry and the results are shown in Fig. 1b. While talking about the cyclic voltammetry results of our synthesized compound 7, in the absence of ss-DNA we noticed two distinct anodic and cathodic peaks with peak potential of 780 mV and 210 mV respectively. The separation between these peak-to-peak potentials (Δ*E*_p_) was 570 mV, represented by the black colored curves 7A, in Fig. 1b. The current of both peaks decreased upon the addition of ss-DNA. Anodic and cathodic peak potentials both were enhanced towards more positive and more negative values, respectively along with increase of peak-to-peak potential. Now the anodic and cathodic peaks potentials become 820 mV and 170 mV respectively, with the separation between these peak-to-peak potentials (Δ*E*_p_) increased to 650 mV, as shown by curve 7a in Fig. 1b.

The formation of a DNA-bound complex with synthesized compounds lead to a decrease in the concentration of unbound synthesized compound and thus a drop in the anodic current, is observed due to the presence of ss-DNA.^48^ The decrease in anodic current is mainly because of the lower rate of diffusion of the DNA-bound species and the decrease in the concentration of unbound synthesized compound as the synthesized compound–DNA complex formed upon addition of DNA. Interaction with ss-DNA also resulted in a shift of the peak potential to a more positive value. According to literature a rise in peak potential suggests an interaction in intercalative mode.^49^ So, while observing the positive shift in the peak potential of the synthesized compound, it is suggested that the synthesized compound is intercalating into the DNA double helix. Next the decrease in peak current of the synthesized compound after the addition of ss-DNA is likely caused by the intercalation of the synthesized compound into the large, slowly diffusing DNA, which results in a significant decreasing in the apparent diffusion coefficient, as per observations.^40^

The term “strong interactions with ss-DNA for biosensor applications” refers to a chemical or material's strong affinity for binding to ss-DNA and its possible effects in the field of biosensor technology. These implications include a number of important points.

Firstly, these interactions can increase the sensitivity of biosensors, allowing for the detection and measurement of specific DNA sequences or chemicals even at low concentrations.^50^ Secondly, strong interactions can improve the selectivity of biosensors, reducing the likelihood of false positives or false negatives by enhancing the ability to differentiate between different DNA sequences or targets.^51^ Additionally, strong interactions can lead to faster response times in biosensors, enabling quick and accurate detection of biological analytes or modifications to DNA structure.^52^ Finally, biosensors with strong interactions with ss-DNA may find a wider range of applications in industries such as biotechnology, environmental monitoring, and medical diagnostics, due to their enhanced performance features.^53^

### 3.3 Computational analysis

**Fig. 2:**
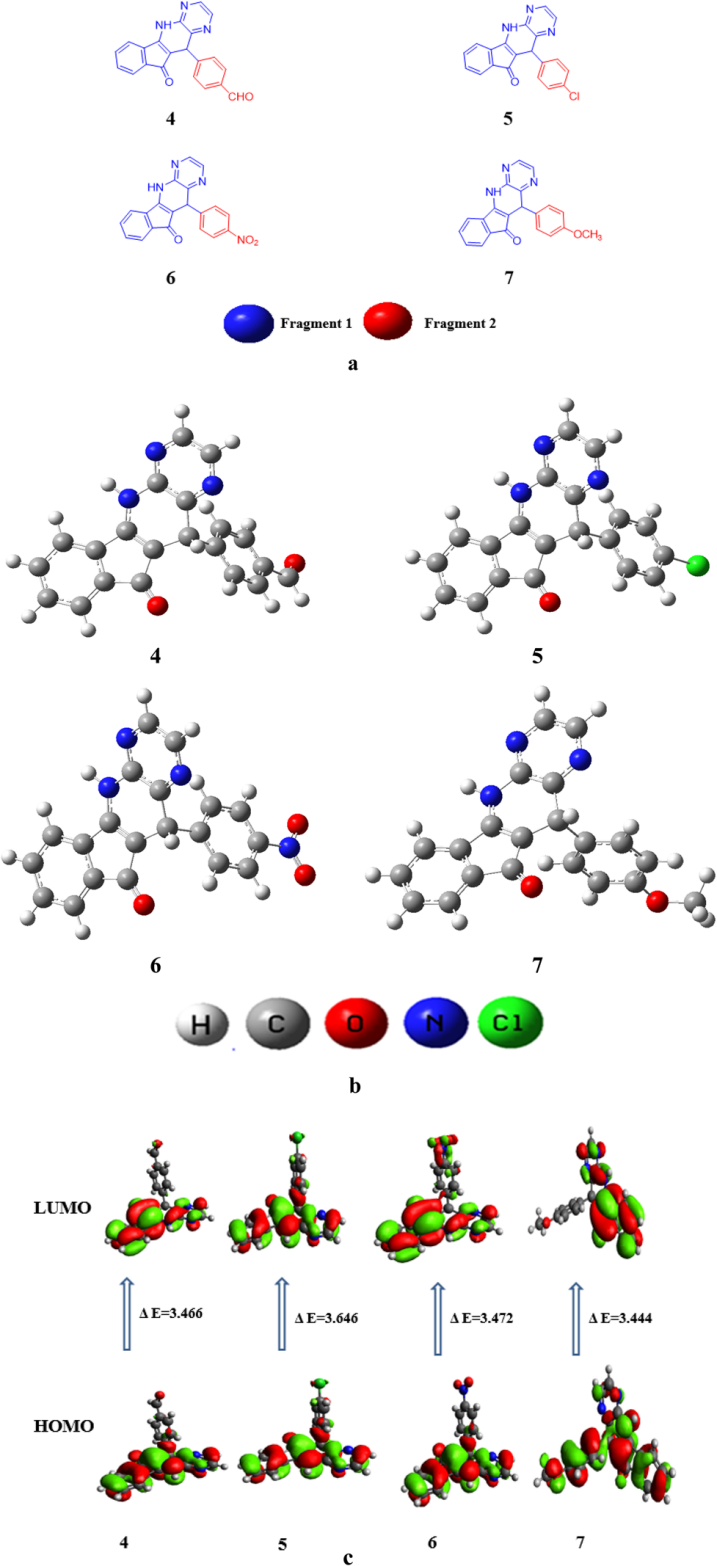
(a) Structures of studied compounds (4–7) (b) optimized structures of investigated compounds (4–7) (c) contour surfaces of FMOs of the studied compounds (4–7).

In this research paper, configuration of investigated compounds (4–7) has two different portions: fragment 1 and fragment 2. In compounds 4, 5 as well as 6 and 7 (5,11-dihydro-10*H*-indeno[2′,1′:5,6]pyrido[2,3-*b*]pyrazin-10-one) acts as a fragment 1 whereas terephthalaldehyde, 4-chlorobenzaldehyde, 4-nitrobenzaldehyde and 4-methoxybenzaldehyde respectively act as a fragment 2. In the context of examined molecular entities, the regions responsible for charge transfer phenomena are depicted in Scheme 1B, whereas the structural representations of the investigated compounds, as generated using ChemDraw, are elucidated in Fig. 2a. Furthermore, their optimized structures are epitomized in Fig. 2b. DFT and TDDFT calculations were performed to investigate the impact of two distinct molecular fragments on their NLO characteristics and the energy gap between molecular orbitals. This study is expected to provide valuable insights for the development of NLO materials with superior performance.

#### 3.3.1 Natural bond orbitals (NBOs) analysis

NBO analysis stands as the preeminent approach renowned for its precision in elucidating the dynamics of electron transfer mechanisms, examining bond interactions, and providing elucidation of hyperconjugative interactions that occur between nucleophilic and electrophilic species.^54^ The utilization of NBO analysis holds promise for illuminating the establishment of equilibrium states within the donor–π–acceptor framework, as well as comprehending the evolution of charge density of fully occupied, covalently bonded, or donor-type orbitals to partially occupied, non-covalently bonded, non-donor-type NBOs.^55^ For each transition donor (i) and acceptor (j), stabilization energy (*E*^(2)^) accompanying with delocalization i → j are computed by the following equation:
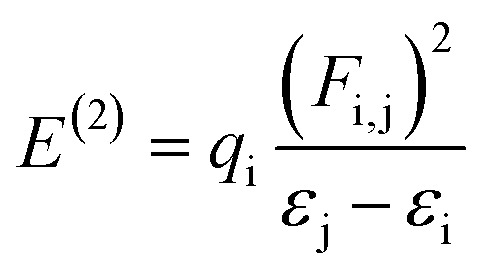


*F*(i,j) indicates the diagonal, *q*_i_ donor orbital occupancy, and *ε*_j_ and *ε*_i_ are off-diagonal NBO Fock or Kohn–Sham medium elements. *E*^(2)^ specifies the stabilization energy^56^ When *E*^(2)^ has a high value and the system as a whole has more conjugation, there is a significant interaction between i and j. The findings show that substituent's capacity to withdraw or donate electrons plays a significant part in causing the atomic charge on the carbon atom connected with substituents. Electronegativity plays a significant part in stabilization because highly electronegative groups, such as nitrogen, chloro, and methoxy group insertion, can promote hyper-conjugation and therefore stabilize the lone pair of atoms in transition states.^57^ The specific nominated values for compounds 4 to 7 in the second-order perturbation approach are listed in Table 1.

**Table 1:** NBOs study for investigated compounds (4–7)

Compounds	Donor(i)	Type	Acceptor(j)	Type	*E* ^(2)^ [*kcal mol*^−1^]	*E*(j) − *E*(i) [*a.u.*]	*F*(i,j) [*a.u.*]
4	C14–C15	π	C26–O27	π*	25.77	0.3	0.079
	C16–C18	π	C14–C15	π*	0.79	0.28	0.014
	C15–C26	σ	C14–N34	σ*	6.54	1.1	0.076
	C28–H29	σ	C30–H31	σ*	0.5	0.97	0.02
	N34	LP(1)	C14–C15	π*	43.81	0.32	0.108
	O38	LP(2)	C37–H39	σ*	23.26	0.63	0.11
5	C14–C15	π	C26–O27	π*	25.78	0.3	0.079
	C16–C17	π	C14–C15	π*	0.86	0.28	0.014
	C5–C6	σ	C4–C14	σ*	5.81	1.15	0.073
	C28–H29	σ	C30–H31	σ*	0.5	0.97	0.02
	N34	LP(1)	C14–C15	π*	43.3	0.32	0.107
	O27	LP(2)	C3–C26	σ*	21.58	0.67	0.109
6	C19–C23	π	N37–O39	π*	27.57	0.15	0.061
	C14–C15	π	C16–C17	π*	0.52	0.31	0.012
	C5–C6	σ	C4–C14	σ*	5.83	1.15	0.073
	C28–H29	σ	C30–H31	σ*	0.5	0.97	0.02
	O38	LP(3)	N37–O39	π*	164.39	0.14	0.14
	O27	LP(2)	C3–C26	σ*	21.52	0.67	0.109
7	C14–C15	π	C26–O27	π*	25.06	0.31	0.078
	C12–N33	π	C16–C18	π*	0.62	0.37	0.014
	C15–C26	σ	C14–N34	σ*	6.66	1.1	0.076
	C3–C26	σ	C14–N34	σ*	0.51	1.08	0.021
	N34	LP(1)	C14–C15	π*	43.45	0.32	0.107
	O27	LP(2)	C3–C26	σ*	21.9	0.67	0.109

NBO study depicts four types of transition *i.e.*, σ → σ*,π → π*, LP → σ* and LP → π*. These transitions are vital for the intramolecular charge transferal and results in the stabilization of system. In π → π* transitions, the charge transference takes place within the molecule is more prominent as compared to σ → σ*. A few transitions are presented in Tables S3–S6.†

In compound 6, the significant π → π* electronic transition which showed the highest stabilization energy value (27.57 kcal mol^−1^) is for π(C19–C23) → π*(N37–O39). While π(C14–C15) → π*(C16–C17) was of the lowermost energy transition having 0.52 kcal mol^−1^ energy. Moreover, σ → σ* transition as σ(C5–C6) → σ*(C4–C14) had 5.83 kcal mol^−1^ as maximum energy and σ(C28–H29) → σ*(C30–H31) having 0.51 kcal mol^−1^ lowest energy transitions. Moreover, LP → π* shows the highest value of 164.39 kcal mol^−1^ for LP3(O38) → π*(N37–O39) transition and LP → σ* had the stabilization energy value of 21.52 kcal mol^−1^ for LP2(O27) → σ*(C3–C26) transitions.

For compound 4, π → π* electronic transition with the maximum value of stabilization energy (25.77 kcal mol^−1^) is shown by π(C14–C15) → π*(C26–O27). Whereas, π(C16–C18) → π*(C14–C15) shows lowest energy transition of 0.79 kcal mol^−1^ energy. In addition, σ → σ* transition like σ(C15–C26) → σ*(C14–N34) has 6.54 kcal mol^−1^ as uppermost energy and σ(C28–H29) → σ*(C30–H31) depicts 0.50 kcal mol^−1^ lowest energy. The LP → π* is perceived at 43.81 kcal mol^−1^ by LP1(N34) → π*(C14–C15), while the σ* value perceived in LP2(O38) to σ*(C37–H39) transition is 23.26 kcal mol^−1^.

For compound 5, the π → π* transition shows the utmost value of stabilization energy at 25.78 kcal mol^−1^ by π(C14–C15) → π*(C26–O27) whereas, π(C16–C17) → π*(C14–C15) consisting of the lowest energy as 0.86 kcal mol^−1^. The σ → σ* transition: σ(C5–C6) → σ*(C4–C14) had 5.81 kcal mol^−1^ highest and σ(C28–H29) → σ*(C30–H31) comprising of the lowest energy as 0.5 kcal mol^−1^. Moreover, LP → π* had the maximum value at 43.3 kcal mol^−1^ for LP1(N34) → π*(C14–C15) while the σ* value (21.58 kcal mol^−1^) is observed for LP2(O27) → σ*(C3–C26) transition. For compound 7, π → π* transition contained highest value of stabilization energy 25.06 kcal mol^−1^ for π(C14–C15) → π*(C26–O27). However, lowest energy (0.62 kcal mol^−1^) was disclosed for π(C12–N33) → π*(C16–C18). Further, σ → σ* transition such as σ(C15–C26) → σ*(C14–N34) comprising highest energy of 6.66 kcal mol^−1^. The σ(C3–C26) → σ*(C14–N34) is found with lowest energy of 0.51 kcal mol^−1^. Moreover, LP → π* has the highest value of 43.45 kcal mol^−1^ due to LP1(N34) → π*(C14–C15) while the σ* value noticed in LP2(O27) → σ*(C3–C26) was 21.9 kcal mol^−1^ transition. Among all the entitled compounds, compound 6 shows the maximum stability due to extended hyper-conjugation with 27.57 kcal mol^−1^. The overall stability order is 6 > 4 > 5 > 7. Therefore, NBO study of these compounds shows that the extended hyper conjugation and strong ICT plays a significant role in stabilizing these compounds and signifies charge transmission properties.

#### 3.3.2 Electronic structure

The preeminent frontier molecular orbitals (FMOs) consist of the highest energy level among occupied molecular orbitals, denoted as the highest occupied molecular orbital (HOMO), and the lowest energy level among unoccupied molecular orbitals, referred to as the lowest unoccupied molecular orbital (LUMO).^58^ FMOs assist in describing the planned molecule's chemical affinities and interactions with other moieties.^59^ It also aids in the deduction of any-electron system's reactive sites.^60–62^ The energy gap of FMOs (*E*_gap_ = *E*_LUMO_ − *E*_HOMO_) clarifies the reactivity, hardness, and softness of molecules as well as their ability to transmit electrons.^63^ Molecules with larger *E*_gap_ values are more stable, have lower chemical affinities, and have higher hardness values, which makes them more likely to interfere with changes in the electrical arrangement.^64^ In contrast, a soft, highly reactive, and unstable molecule has a smaller *E*_gap_ value. Additionally, *E*_gap_ is thought of as a fundamental feature for examining NLO qualities. In scientific terminology LUMO is conceptualized as the orbital displaying partial electron occupancy and possessing electron-accepting properties. Conversely, HOMO is located within an orbital exhibiting an inclination towards electron donation.^65^ The NLO behavior can be increased by the narrowed HOMO–LUMO energy gap, which is highly polarizable.^66^ Compounds 4 to 7 were subjected to an FMO analysis, with the findings presented in Table S7.†

Table S7† shows that the *E*_HOMO_/*E*_LUMO_ values for compounds (4–7) are obtained as −6.131/−2.665, −6.068/−2.604, −6.261/−2.789, and −5.858/−2.414 eV, correspondingly. The *E*_LUMO_ − *E*_HOMO_ values of titled compounds are calculated as 3.477, 3.646, 3.472 and 3.444 eV, respectively. Table S7† describes that the compound 7 has lowest band gap value than all other derivatives. This might be due to the presence of methoxy group at the *para* position of benzene ring that have positive inductive effect and improve the charge transference in molecule. The *E*_gap_ is indispensable for charge transfer process, lesser the band gap greater will be the transference of charge. The contour surface diagrams of HOMO and LUMO are presented in Fig. 2c. It represents that HOMO is mainly located over the [5,11-dihydro-10*H*-indeno[2′,1′:5,6]pyrido[2,3-*b*]pyrazin-10-one] part of compound 4. While, in LUMO the charge density is majorly concentrated on [5,11-dihydro-10H-indeno[2′,1′:5,6]pyrido[2,3-*b*]pyrazin-10-one] moiety and some effects are on oxygen atom of benzaldehyde moiety. In 5 compound the charge density for HOMO is positioned over 5,11-dihydro-10H-indeno[2′,1′:5,6]pyrido[2,3-*b*] pyrazin-10-one and small effect on chlorine atom of chlorobenzen moiety, Whereas, in LUMO the charge density is concentrated on the same portion like HOMO.

In compound 6 the charge density for HOMO is situated on 5,11-dihydro-10H-indeno [2′,1′:5,6] pyrido[2,3-*b*]pyrazin-10-one, while at LUMO the charge density additionally observed at nitro group of nitrobenzene. Similarly, for compound 7 charge density for HOMO is located over 3-amino-2-(4-methoxybenzyl)-1*H*-inden-1-one part and small effect are observed at pyrazine part, while at LUMO charge density observed at 3-amino-2-(4-methoxybenzyl)-1*H*-inden-1-one part are displayed in Fig. 2c. The values of HOMO−1, LUMO+1, HOMO−2 and LUMO+2 is exhibited in Table S7† and their FMOs diagrams are provided in Fig. S1.†

#### 3.3.3 Density of states (DOS) analysis

The electronic characteristics of all the examined compounds are estimated using the DOS data to support the FMO analysis. According to Fig. S2,† the electric density of charges decentralized across HOMO and LUMO in various patterns. The electron distribution is shown by the DOS, moving from HOMO with a strong tendency to donate electrons to LUMO with a strong tendency to gain electrons.^67^ By calculating the DOS fractions on HOMO and LUMO, the charge dispersion scheme on molecular orbitals (MOs) based on the different portions was confirmed.^68^ In DOS pictographs, the HOMO is represented by negative numbers, whilst the LUMO is represented by positive values along the *x*-axis. The *E*_gap_ is the difference between the two.^69^ We divided the entitled molecules into discrete parts for the DOS computation. Compounds 4 to 7 are separated into two fragments *i.e.*, fragment 1 and fragment 2. The electronic cloud of HOMO in compound 4 is only arranged on fragment 1 whereas, small effect on fragment 2 are observed for LUMO as illustrated in Fig. 2c. In compound 5 the same pattern of HOMO and LUMO concentration is noticed *i.e.*, major electronic cloud on fragment 1. While, in compound 6 and 7 a different pattern of HOMO and LUMO concentration is observed *i.e.*, major electronic cloud on fragment 1 and the small effect are observed on fragment 2. In DOS spectra, the highest charge density of HOMO is positioned over fragment 1 as red peak traced at approximately −2.5 eV, however the electronic charge distribution for LUMO is observed to be at 1.5 to 2 eV on fragment 2. It is perceived that fragment 1 contributes 94.7% to HOMO and 97.3% to LUMO in compound 4 while, contribution of fragment 2 is 5.3 and 2.7% to HOMO and LUMO, correspondingly. However, for fragment 1, 92.7, 95.3 and 52.6% contribution to HOMO while, 99.0, 92.9 and 99.5% to LUMO is shown in compounds 5, 6 and 7, respectively. Whereas 7.3, 4.7 and 47.4% contributions to HOMO while, 1.0, 7.1 and 0.5% LUMO are shown by fragment 2, respectively. From the above discussion, it is clear that the HOMO of all the derivatives are located on the fragment 1 whereas, the LUMO are extensively resided on the fragment 2 (see Fig. S2 and Table S8†). In general, DOS study demonstrates substantial transferal of charge from fragment 1 to fragment 2 in all the examined compounds.

#### 3.3.4 Global reactivity parameters (GRPs)

To calculate the GRPs such as electron affinity (EA), electronegativity (*X*), global electrophilicity index (*ω*), ionisation potential (IP), global softness (*σ*), global hardness (*η*), and the chemical potential (*μ*),^63,70–73^ the HOMO and LUMO energy gap values are crucial. All these parameters were calculated by utilizing following eqn (5)–(12) (ref. 74 and 75) and their values are demonstrated in Table S9.†

5 IP = −*E*_HOMO_

6 EA = −*E*_LUMO_

7
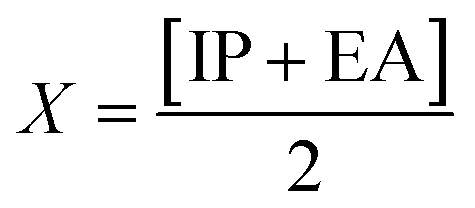
8
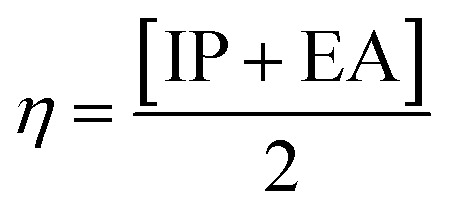
9
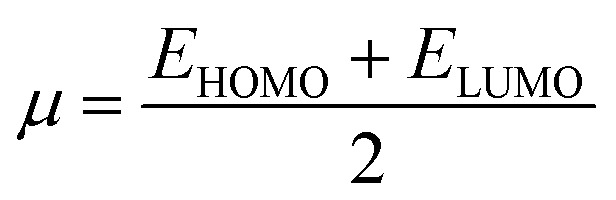
10
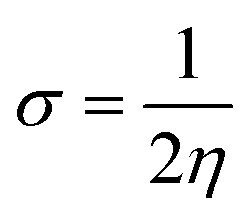
11
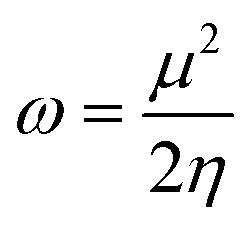
12
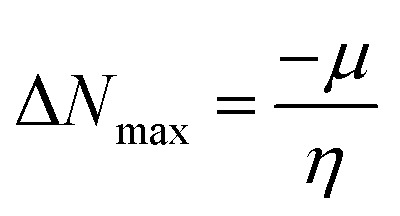


The movement of electrons within a compound is designated as the value of *μ*,^76^ whereas the chemical reactivity and stability is determined by *η*.^77^ When a molecule gains an extra electronic charge from an external source,^78^ this determines its stabilisation energy *ω*. The date in Table S9† shows the GRPs of titled chromophores at B3LYP functional. The energy gap is directly affected by the chemical potential (*μ*) and hardness (*η*) parameters. The negative findings of chemical potential (*μ*) indicates that the compound under study is sufficiently stable. The *E*_gap_, hardness, stability and chemical potential exhibit direct relation with each other, whereas inverse relation with reactivity.^57^ The *η* values of these compounds are 1.733, 1.732, 1.736 and 1.722 eV. Compound 6 exhibits the highest stability owing to the greatest hardness value (1.736 eV). Whereas *σ* values are 0.288, 0.288, 0.288 and 0.290 eV^−1^. The smaller hardness and larger softness values of compound 7 in comparison to others show its higher reactivity and less stability due to lower *E*_gap_ of compound 7. The outcomes revealed that compound 7 is highly polarizable and demonstrates exceptional NLO response.

#### 3.3.5 Transition density matrix (TDM) analysis

**Fig. 3:**
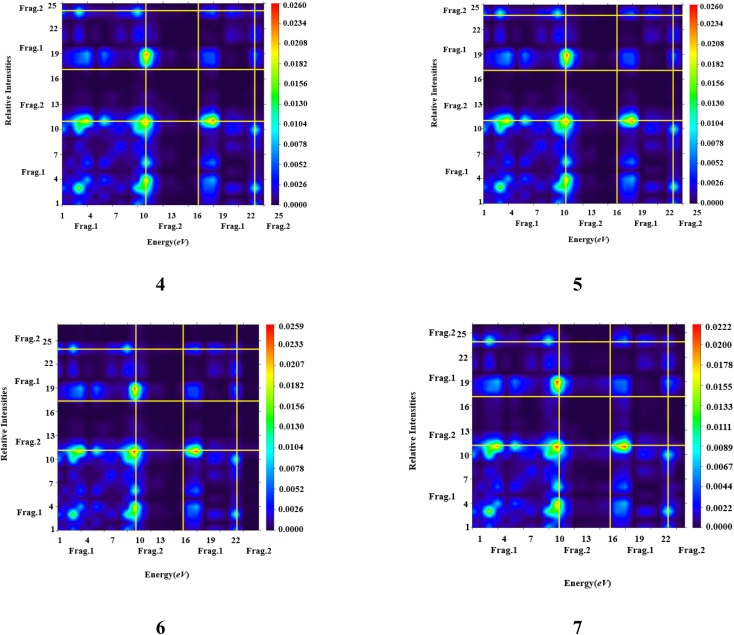
TDM heat maps of compound (4–7).

In order to evaluate and explain electronic excitation events in molecular systems, TDM is a useful technique. For every change between two eigen states in a many system, the TDM analysis offers a distinctive spatial heat map that depicts the scattering of related electron–hole pairs and enables one to determine their decentralization and resonance durations.^79^ We separated our compounds into two parts, fragment 1 and fragment 2, for TDM investigation. The hydrogen atoms are not significantly engaged in the transitions in our investigation; therefore, their influence has been ignored. At this level of DFT, the TDM ends of compounds 4 to 7 are derived, and Fig. 3 shows their pictographs. According to the FMOs study, the charge density is mostly applied to fragment 1, which produced a substantial change in TDM pictographs. The charge coherent is efficiently contained on fragment 1 and extended diagonally towards fragment 2, as seen in Fig. 3. Additionally, it appears that excitation of coherence and electron hole pair creation both spread non-diagonally across the whole TDM map. The results of TDM snapshots show that the charge separation from the ground state (S_0_) to the excited state (S_1_) is easier and more rapid. On the other hand, estimates of TDM heat maps for compounds 4 to 7 indicate that exciton separation in the excited state might be simplified and improved for use in future applications.

#### 3.3.6 UV-visible analysis

UV-visible analysis was done in the gas phase using TD-DFT calculations at the B3LYP level to explain charge transfer within the synthesised molecules and demonstrate their absorption properties.^80^ The calculated outcomes of transition energy (*E*), absorption wavelength (*λ*_max_), transition moment, oscillator strength (*f*_os_), and transition nature of the synthesize compounds (4–7) were presented in Table S11 and Fig. S3.†

It is apparent from the data presented in Table S11† that there exists an inverse correlation between transition energy outcomes and the maximum absorption wavelengths (*λ*_max_). As the transition energy of molecules escalates *λ*_max_ decreases. The computed values of *λ*_max_ for all synthesised compounds are in the range of 310.131–443.704 nm. The lowest value of *λ*_max_ is examined in 5 (310.131 nm) due to poor charge transference as chloro has electron withdrawing effect it withdraws electrons from fragment 1. Charge transference was improved in 6 (315.417 nm) due to nitro group which withdraws electrons. Compound 7 with *λ*_max_ (443.704 nm) exhibits maximum absorption wavelength due to methoxy group as it donates electron to fragment 1 and charge is efficiently transferred to fragment 1 (Tables S12–S15†). In summary, the comprehensive discourse supports the notion that all chemical compounds can be regarded as adept candidates for NLO materials. However, it is noteworthy that compound 7 exhibits the potential for heightened proficiency in this context, attributable to its diminished band gap energy (*E*_gap_) and augmented values for the maximum wavelength (*λ*_max_).

#### 3.3.7 FT-IR analysis

The vibrational bands of compound (4–7) were examined using B3LYP/6-31G (d,p) DFT level. The experimental and theoretical values of FT-IR absorption for entitled compounds can be seen in Tables S16–S19.†

##### 3.3.7.1 C–H vibrations

In the aromatic ring stretching bands related to C–H vibrational are present at 3100–3000(cm^−1^).^81^ In the current study, we got computational calculations for C–H of compound 4 at 3194–3186 cm^−1^, which were enough close to the observed vibrational band 3214 cm^−1^. Similarly, compounds 5, 6 and 7 were obtained at range at 3193–3160, 3224–3174 and 3203–3130 cm^−1^ which were very close to the experimental vibrational bands 3193, 3347 and 4359 cm^−1^. A reasonable consistency has been observed between DFT based and experimentally determined FT-IR values. Further, the calculated in-plane bending vibrational wavenumbers for C–H bond appeared 1474 to 892 cm^−1^ for all studied compounds as reported in Tables S16–S19.†

##### 3.3.7.2 C–C vibrations

The stretching frequencies of C=C are shown at 1650–1400 (cm^−1^).^82^ The measured C–C vibrational modes are observed at 1665–1433 cm^−1^ for compounds 4 to 7. Absorption bands which are measured clearly matching to experimental bands of 1660, 1661, 1659 and 1658 cm^−1^ for 4–7 compounds respectively as can be seen in Tables S16–S19.†

##### 3.3.7.3 C=N vibrations

The values for the C=N vibrations were mentioned at 1585, 1545, 1544 and 1544 cm^−1^ for compounds 4–7 respectively. However, some other vibrations are observed at 1600 cm^−1^ range for all studied compounds between the carbon and nitrogen of pyridine ring.

##### 3.3.7.4 N–H vibrations

Usually, the N–H wavenumbers lie in the span of 3450–3250 (cm^−1^).^83^ In the current work, the N–H stretching bands simulated, are observed at 3614, 3614, 3613 and 3615 cm^−1^ in compounds 4–7 respectively. However, some other vibrations in-plane bending vibrations are observed at 892 cm^−1^ for all investigate compounds can be seen in Tables S16–S19.†

##### 3.3.7.5 Other vibrations

In our studied compounds 4–7 different substituents are attached with fragment 2 showing different vibrations. However, some other calculated vibrations of CHO, C–Cl, NO_2_ and OCH_3_ were observed at 892, 770, 771 and 772 cm^−1^ which were very close to that of experimental vibrational band 843, 759, 765 and 720 cm^−1^ as represented in Tables S16–S19.†

#### 3.3.8 Natural population analysis (NPA)

The atomic charges for 4 to 7 were calculated by using NBO analysis. The charge dissemination of 4 to 7 are depicted in Fig. S4.† The charge distribution demonstrates that all oxygen atoms have negative charges which were coupled with hydrogen and carbon atoms, but carbon and hydrogen carried a high positive charge for entitled compounds. Moreover, for compound 4 all nitrogen atoms and that of carbon atoms, *i.e.*, N32, N33 and N34 and C1, C2, C3, C4, C5, C6, C11, C12, C15, C16, C17, C18, C19, C21, C23 and C30, carried negative charges, whereas some carbon atoms like C13, C14, C26, C28, and C37 were positively charged. Likewise, for compound 5 all nitrogen atoms and carbon atoms, *i.e.*, N32, N33 and N34 and C1, C2, C3, C4, C5, C6, C11, C12, C15, C16, C17, C18, C23 and C30, carries negative charges, while some carbon atoms like C13, C14, C19, C21, C26 and C28, are positively charged. All nitrogen and carbon atoms *i.e.*, N32, N33 and N34 and C1, C2, C3, C4, C5, C6, C11, C12, C15, C16, C17, C18, C19, C21 and C30 carries negative charges, while some carbon atoms like C13, C14, C23, C26 and C28 are positively charged for compound 6. For compound 7 all nitrogen atoms and carbon atoms *i.e.*, N32, N33 and N34 and C1, C2, C3, C4, C5, C6, C11, C15, C16, C17, C18, C19, C21 and C38, carries negative charges, whereas some carbon atoms like C12, C13, C14, C23, C26, C28 and C30 are positively charged Moreover, all hydrogen atoms holds positive charge for compounds 4–7 as shown in Fig. S4.† In compound 6, oxygen atom (O38) bonded with nitrogen (N37) atoms are positively charged and in compound 7 oxygen atom (O37) bonded with carbon (C38) atom possess negative charge as shown in Fig. S4.†

#### 3.3.9 Nonlinear optical (NLO) properties

Nonlinear optics is currently acknowledged as the most fascinating subject to be explored in various scientific fields.^84,85^ NLO products are extensively used in optoelectronic devices, signal processing and communication networking^86^ the electrical characteristics of molecule, which are successively linked to the polarizability 〈*α*〉, and nonlinear responses: first hyper polarizability (*β*_tot_) and second hyper polarizability 〈*γ*〉, determine how the molecule will behave optically. The electronic densities in an extended conjugated system respond to an applied external electric field.^87^ Thus, it modifies their dipole moments. For the entitled molecules (4–7), the NLO parameters such as dipole moment (*μ*), Average polarizability 〈*α*〉, second-order polarizability (*β*_tot_), and second order hyper-polarizability 〈*γ*〉 values were computed *via* utilizing B3LYP/-311G(d,p) functional and the major results are exhibited in Table S20† while their major contributing tensors are detailed discussed in Tables S21–S24.†

Among all the entitle compounds, the greatest value of *μ*_tot_ is observed in 6 which may be due to the existence of highly electronegative nitro group. The decreasing order of dipole moments of computed molecules is: 6 > 4 > 5 > 7. Thus, it is evident from the results that ICT and electron transport rate increase with increasing *μ*_tot_ value. The results of 〈*α*〉 of all the compounds are found comparable with one another. The compound 7 unveiled the highest value of 〈*α*〉 which is 3.90 × 10^−23^ esu which may be owing to the inductive effect of methoxy group at para position while, 5, shows the lowermost 〈*α*〉 value of 3.78 × 10^−23^ esu. Among these newly synthesize compounds, the order of 〈*α*〉 for all studied compounds is as: 7 > 4 > 6 >5.

The charge transfer within the molecule is associated with the first hyper-polarizability. All of the titled compounds (4–7) exhibit the strongest ICT. The greater the ICT, greater the NLO reaction would be. The *β*_tot_ value of 6 is examined to be 9.20 × 10^−30^ esu which is the lowermost observed value, while 7is found to have the utmost value (15.6 × 10^−30^ esu) of *β*_tot_ among all the studied molecules. The *β*_tot_ values of 4–7 is observed to be 7.88 × 10^−30^, 9.74 × 10^−30^, 9.20 × 10^−30^, and 15.6 × 10^−30^ esu, respectively. The *β*_tot_ values were found to be in the declining order as: 7 > 5 > 6 > 4. For the investigation of hyper-polarizability characteristics, Para-nitro aniline (PNA) is used as the reference molecule (*β*_tot_ = 0.3610 × 10^−30^ esu).^88^ By comparing our results with PNA molecule, we determined that the hyper-polarizability values of 4 to 7 are 21, 26, 25 and 43 times larger than PNA molecule, respectively.

Two-photon absorption (TPA) phenomena in NLO materials are thought to be the basis for the second hyper-polarizability of the compounds. As shown in Tables S21–S24,† a substantial second-order hyper polarizability response was perceived along the *x*-axis in all the studied compounds, with 7 having the largest value of 〈*γ*〉 among the derivatives. The decreasing order of 〈*γ*〉 of the above-mentioned compounds is as follows: 7 > 6 > 4 > 5. From the above explanation, it should be clear that different kinds of substituents played a crucial role in exhibiting amazing NLO amplitude.^89^

### 3.4 Biological evaluation

#### 3.4.1 *In vitro* anti-oxidant activity

The antioxidant activity of nitrogen-containing heterocyclic derivatives of pyrido[2,3-*b*]pyrazine were measured by a reported method^41^ which was used as tool to measure the DPPH radical scavenging activity.^90^ The fundamental concept underlying this technique involves the diminution of the chromatic intensity, specifically within the purple-violet spectral range, through the introduction of a test substance that serves as a donor of hydrogen atoms within the provided DPPH solution. The outcome entails the generation of the reduced state of the initially radical DPPH molecule from its non-radical counterpart. Quantitative antioxidant measurement was done by UV-vis spectrophotometer at wavelength 517 nm. Ascorbic acid, a well-known antioxidant, was employed as a positive control in the experiment for comparison.

**Fig. 4:**
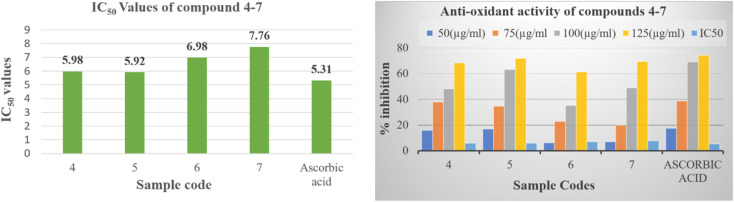
*In vitro* antioxidant activity of compounds (4–7) and ascorbic acid reference.

The empirical findings pertaining to the antioxidative efficacy of the tested compounds, in conjunction with the reference pharmaceutical agent, are displayed within Table S25† for reference. The purpose of this work is to identify the potential pyrido[2,3-*b*]pyrazine based heterocyclic compound as antioxidant agent. The results given therein indicate that all the synthesized compounds (4–7) demonstrated potent to moderate free radical scavenging activity or inhibition. Compounds 5 proved to be potent DPPH scavengers, as it exhibited antioxidant activity with IC_50_ values 5.92 ± 0.11 μM in comparison to the standard antioxidant, which showed IC_50_ values of 5.31 ± 1.22. While the compounds 4, 6 and 7 showed moderate activity ranging 5.98 ± 1.95 to 7.76 ± 1.76 μM (Fig. 4). The order of decreasing antioxidant activity is 5 > 4 > 6 > 7. Further the analysis of Table S25† indicated that radical scavenging activity in DPPH method increases with increasing concentration.

#### 3.4.2 *In vitro* antiurease activity

All the synthesized pyrido[2,3-*b*]pyrazine based heterocyclic compounds (4–7) were also evaluated for urease inhibitory action. Thiourea was used as a reference point to assess the effects of substituents about the pyrido[2,3-*b*]pyrazine based scaffold on their urease inhibitory potential. To compute the IC_50_, standard and synthesized compounds (4–7) were tested at different concentrations under the same conditions. The results collected in Table S26† revealed that all of these prepared compounds (4–7) tested for urease inhibitory capability in the current bioassay displayed enzymatic inhibition. From these, compound 5 proved to be potent inhibitors exhibiting IC_50_ values 4.12 ± 1.18 μM. The remaining compounds, 4, 6, and 7, showed different degrees of inhibitory action, with IC_50_ values ranging from 4.77 ± 0.92 μM to 11.91 ± 0.34 μM.

## 4. Conclusion

Four Pyrido[2,3-*b*]pyrazine based heterocyclic compounds (4–7) were synthesized and evaluated for their electrochemical DNA sensing, computational studies and biological activities. Cyclic voltammetry is used to detect electrochemical DNA sensing, showing that interaction of compound (4–7) with ssDNA is in intercalative mode. These results indicated that Pyrido[2,3-*b*]pyrazine based heterocyclic compounds (4–7) demonstrated significant potential for DNA sensing under optimised conditions and can be employed for sensing applications of different biomolecules with minor modifications. In DFT study, NBO findings revealed that effective CT is possible within the molecules. The FMOs exploration designates the phenomena of charge transfer and chemical reactivity in 4–7 compounds. With the assistance of FMO energies, GRP has been also calculated that showed that compound 7 with lower band gap (3.444 eV), exhibited smaller hardness (1.722 eV) with larger value of softness which indicated its larger polarizability. Additionally, compound 7 showed less energy gap and remarkable NLO response with highest 〈*α*〉, *β*_tot_ and 〈*γ*〉 values observed as 3.90 × 10^−23^, 15.6 × 10^−30^ and 6.63 × 10^−35^ esu, respectively. Comparative analysis with P-NA molecule showed that the fused ring heterocyclic pyrazin based heterocyclic compounds act as efficient NLO materials and can be used in progressive photonic applications. Furthermore, the novel compound (4–7) showed encouraging antioxidant and urease inhibitory properties, highlighting their potential as therapeutic agents.

## Conflicts of interest

There are no conflicts of interest to declare.

## Supplementary Material

RA-013-D3RA05365B-s001
