# Supplementary material for: Multicomponent synthesis of pyrido[2,3-b]pyrazine derivatives: electrochemical DNA sensing, nonlinear optical properties and biological activity
Source: RSC Adv. 2023 Nov 1;13(46):32160–74. doi: 10.1039/d3ra05365b (PMC10619479; doi:10.1039/d3ra05365b)
Supplement: RA-013-D3RA05365B-s001 [file RA-013-D3RA05365B-s001.pdf]

Electronic Supplementary Material (ESI) for RSC Advances.
This journal is © The Royal Society of Chemistry 2023

Supplementary information

Multicomponent Synthesis of Pyrido[2,3-*b*]pyrazine Derivatives: Electrochemical DNA Sensing, Nonlinear Optical Properties and Biological Activity

Muhammad Rashid[a], Muhammad Khalid[b,c], Abida Ashraf[a,d], Tahira Saleem[a], Iqra Shafiq[b,c] Muhammad Azeem Shakil[a], Briha Zainab[a], Attalla F. El-kott[e,f], Muhammad Yaqub[a] and Zahid Shafiq[a]

[a] Institute of Chemical Sciences, Bahauddin Zakariya University, Multan 60800, Pakistan

[b]Institute of Chemistry, Khwaja Fareed University of Engineering & Information Technology, Rahim Yar Khan, 64200, Pakistan

[c]Centre for Theoretical and Computational Research, Khwaja Fareed University of Engineering & Information Technology, Rahim Yar Khan, 64200, Pakistan

[d]Department of Chemistry, Govt. Graduate College Shah Rukne- Alam Multan

[e]Department of Biology, College of Science, King Khalid University, Abha, Saudi Arabia.
[f]Department of Zoology, College of Science, Damanhour University, Egypt

Corresponding Authors

Dr. Muhammad Yaqub (mayaqub2@yahoo.com)

Dr. Zahid Shafiq (zahidshafiq@bzu.edu.pk, Tel. +92-3006559811)

Table of contents

**1-**	Optimization of reaction conditions for the synthesis of compound 7	**Table S1**
**2-**	Synthesized 2-aminopyrazine derived fused ring heterocyclic compounds **(4-7)**	**Table S2**
**3-**	General mechanism for synthesis of (**4-7**)	**Scheme S1**
**4-**	NBO table of compound (**4-7**)	**Table S3**
**5-**	HOMO-1, LUMO+1, HOMO-2 and LUMO+2 of the studied compounds **4-7**	**Figure S1**
**6-**	Computed energies (*E*) and energy gap (E_{LUMO}-E_{HOMO}) of HOMO and LUMO of investigated compounds	**Table S7**
**7-**	Calculated energies (*E*) and energy gap (ΔE) of HOMO-1, LUMO+1, HOMO-2 and LUMO+2 for **D4-D7.**	**Table S8**
**8-**	Global reactivity parameters of (**4-7)** compounds.	**Table S9**
**9-**	DOS graphs of **4-7** compounds	**Figure S2**
**10-**	DOS %age of HOMO and LUMO for **4-7** compounds	**Table S10**
**11-**	Absorption spectra of compounds 4-7 in gaseous phase	**Figure S3**
**12-**	UV-Visible analysis of compound **4-7**	**Table S11-S14**
**13-**	Calculated vibrational frequencies of **4-7**	**Table S16-S19**
**14-**	NPA graphs of **4-7** compounds	**Figure S4**
**15-**	Computed average polarizability <α> dipole moment (μ_{tot}), first hyperpolarizability (βtot) and second hyperpolarizability (γ_{tot}) of compounds **4-7**.	**Table S20**
**16-**	Dipole polarizability and major contributing tensor (*e.s.u.*) of the studied comp. **4-7**.	**Table S21**
**17-**	Frequency dependent Second hyperpolarizability (*e.s.u.*) of studied compounds **4-7**	**Table S22**
**18-**	The computed first hyperpolarizability (β_{tot}) and major contributing tensors (e.s.u) of compounds **4-7**.	**Table S23**
**19-**	Frequency dependent First hyperpolarizability (*e.s.u.*) of studied compound **4-7**.	**Table S24**
**20-**	Characterization by NMR & FTIR **4-7**.	
**21-**	*In Vitro* Antioxidant Activity of synthesized compounds **(4-7)**	**Table S25**
**22-**	Urease Inhibitory Activity of synthesized compounds **(4-7)**	**Table S26**

Table S1: Optimization of reaction conditions for the synthesis of compound 7.

Entry	Solvent	Catalyst (mol %)	Time (h)	Yield[a] (%)
1	H_2O	No	24	[b]
2	H_2O	p-TsOH	24	[b]
3	Et-OH	Et_3N	24	35
4	Et-OH	L-Proline	24	45
5	Et-OH	AcOH	24	70
6	Et-OH	$InCl_3$	12	60
7	Et-OH	H_2SO_4	09	46
8	Et-OH	p-TSA	09	**89**
9	CH_3CN	p-TSA	09	65
10	CH_3CN	L-Proline	09	72
11	DMF	p-TSA	09	55
12	THF	$InCl_3$	09	45
13	CH_2Cl_2	p-TsOH	09	42
14	CH_2Cl_2	DABCO	09	Trace

[a] Isolated yield [b] No product formed

Table S2: Synthesized 2-aminopyrazine derived fused ring heterocyclic compounds **(4-7)**.

Entry	Product	Ar-CHO	Structure of Product (4-7)	M.P. (°C)	Yield (%)
1	4	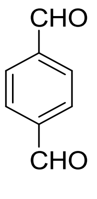	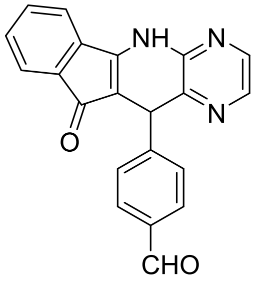	180	82

2	5	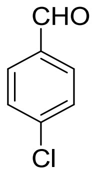	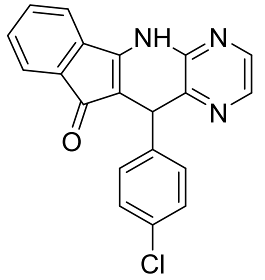	168	82
3	6	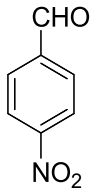	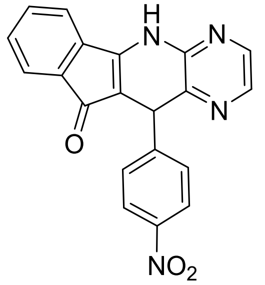	185	85
4	7	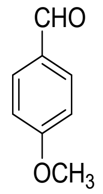	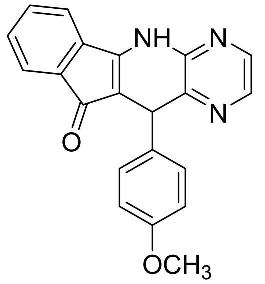	178	89

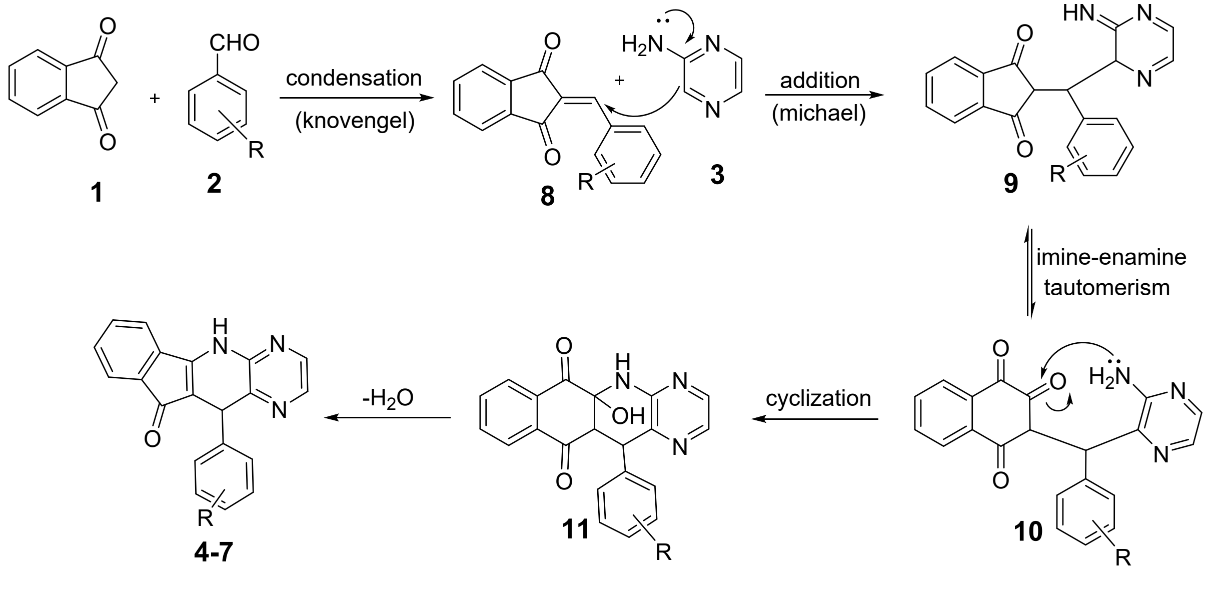

Scheme S1: General mechanism for synthesis of (**4-7**).

Table S3: NBO table of compound **4**.

**Donor(*i*)**	**Type**	**Acceptor(*j*)**	**Type**	***E*(2)[a]**	***E*(J)E(i)[b]**	**F(i,j)[c]**
C13-N32	π	C28-C30	π*	24.07	0.34	0.081
C16-C18	π	C21-C23	π*	23.4	0.28	0.072
C17-C19	π	C16-C18	π*	22.14	0.29	0.072
C2-C3	π	C4-C5	π*	21.31	0.29	0.07
C12-N33	π	C28-C30	π*	21.5	0.33	0.075
C28-C30	π	C12-N33	π*	20.4	0.28	0.067
C12-N33	π	C13-N32	π*	19.15	0.3	0.07
C4-C5	π	C1-C6	π*	18.91	0.29	0.067
C13-N32	π	C12-N33	π*	15.82	0.33	0.065
C2-C3	π	C26-O27	π*	14.83	0.29	0.06
C14-C15	π	C4-C5	π*	13.66	0.3	0.06
C37-O38	π	C21-C23	π*	5.09	0.41	0.045
C26-O27	π	C14-C15	π*	4.63	0.4	0.041
C5-C6	σ	C4-C14	σ*	5.82	1.15	0.073
C5-H9	σ	C3-C4	σ*	4.69	1.09	0.064
C3-C26	σ	C4-C5	σ*	4.35	1.2	0.065
C5-C6	σ	C4-C5	σ*	4.04	1.28	0.064
C37-H39	σ	C19-C23	σ*	3.98	1.1	0.059
C11-C12	σ	C30-N33	σ*	3.74	1.15	0.059
C19-C23	σ	C17-C19	σ*	3.16	1.28	0.057
C11-C12	σ	C13-N32	σ*	2.81	1.14	0.051
C21-C23	σ	C19-H24	σ*	2.21	1.15	0.045
C15-C26	σ	C11-C15	σ*	2.13	1.07	0.042
C11-C16	σ	C16-C18	σ*	1.99	1.19	0.044
C13-N34	σ	C12-C13	σ*	1.58	1.33	0.041

C28-N32	σ	C30-H31	σ*	1.44	1.27	0.038
C16-C17	σ	C17-H20	σ*	1.36	1.14	0.035
C12-N33	σ	C30-N33	σ*	1.33	1.38	0.038
C3-C4	σ	C3-C26	σ*	1.03	1.09	0.03
C6-H10	σ	C5-H9	σ*	0.64	0.95	0.022
C2-H8	σ	C1-C2	σ*	0.56	1.07	0.022
N34	LP(1)	C13-N32	π*	42.85	0.27	0.098
O27	LP(2)	C15-C26	σ*	19.12	0.72	0.106
O38	LP(1)	C23-C37	σ*	1.91	1.14	0.042
O38	LP(1)	C37-H39	σ*	0.51	1.06	0.021

Table S4: NBO table of compound **5**.

**Donor(*i*)**	**Type**	**Acceptor(*j*)**	**Type**	**E(2)[a]**	**E(J)E(i)[b]**	**F(i,j)[c]**
C13-N32	π	C28-C30	π*	24.11	0.34	0.081
C16-C17	π	C19-C23	π*	22.47	0.27	0.07
C18-C21	π	C19-C23	π*	21.79	0.27	0.069
C12-N33	π	C28-C30	π*	21.55	0.32	0.075
C2-C3	π	C4-C5	π*	21.32	0.29	0.07
C4-C5	π	C2-C3	π*	17.97	0.31	0.067
C1-C6	π	C4-C5	π*	18.14	0.29	0.065
C2-C3	π	C1-C6	π*	18.69	0.28	0.066
C19-C23	π	C18-C21	π*	19.45	0.3	0.069
C16-C17	π	C18-C21	π*	20.27	0.29	0.068
C28-C30	π	C13-N32	π*	17.73	0.26	0.062
C14-C15	π	C4-C5	π*	13.78	0.3	0.06
C26-O27	π	C14-C15	π*	4.64	0.4	0.041
C26-O27	π	C2-C3	π*	4.31	0.41	0.04
C14-C15	π	C14-C15	π*	3.01	0.31	0.028
C30-H31	σ	C12-N33	σ*	5.16	1.07	0.066
C4-C14	σ	C11-C15	σ*	4.77	1.08	0.064
C21-H25	σ	C19-C23	σ*	4.46	1.08	0.062
C30-N33	σ	C11-C12	σ*	4.02	1.22	0.063
C30-H31	σ	C28-N32	σ*	3.98	1.06	0.058
C13-N32	σ	C12-C13	σ*	3.66	1.37	0.064
C11-C15	σ	C4-C14	σ*	2.96	1.07	0.05
C13-N34	σ	C28-N32	σ*	2.43	1.34	0.051
C11-C12	σ	C12-N33	σ*	2.04	1.16	0.044
C12-C13	σ	N34-H35	σ*	1.99	1.11	0.042
C11-C16	σ	C11-C12	σ*	1.46	0.99	0.034
C16-C18	σ	C11-C12	σ*	1.27	1.07	0.033

C5-C6	σ	C5-H9	σ*	1.07	1.12	0.031
C26-O27	σ	C14-C15	σ*	0.55	1.68	0.027
C12-C13	σ	C11-C16	σ*	0.51	1.13	0.022
C3-C26	σ	C14-N34	σ*	0.5	1.08	0.021
N34	LP(1)	C13-N32	π*	42.78	0.27	0.098
Cl37	LP(3)	C19-C23	π*	12.03	0.33	0.062
Cl37	LP(1)	C21-C23	σ*	1.61	1.48	0.044
N33	LP(1)	C13-N34	σ*	0.52	0.76	0.018

Table S5: NBO table of compound **6**.

**Donor(*i*)**	**Type**	**Acceptor(*j*)**	**Type**	**E(2)[a]**	**E(J)E(i)[b]**	**F(i,j)[c]**
C14-C15	π	C26-O27	π*	25.9	0.3	0.079
C16-C17	π	C19-C23	π*	24.58	0.27	0.074
C13-N32	π	C28-C30	π*	24.1	0.34	0.081
C18-C21	π	C16-C17	π*	22.66	0.29	0.072
C12-N33	π	C28-C30	π*	21.37	0.33	0.075
C16-C17	π	C18-C21	π*	17.73	0.29	0.065
C13-N32	π	C12-N33	π*	15.88	0.33	0.065
C4-C5	π	C14-C15	π*	14.14	0.3	0.059
C14-C15	π	C4-C5	π*	13.52	0.31	0.059
N37-O39	π	N37-O39	π*	7.55	0.32	0.053
C26-O27	π	C14-C15	π*	4.62	0.4	0.041
C26-O27	π	C2-C3	π*	4.29	0.41	0.04
N37-O39	π	C19-C23	π*	4.11	0.46	0.043
C14-C15	π	C14-C15	π*	3.11	0.31	0.028
C16-C17	π	C14-C15	π*	0.78	0.28	0.014
C4-C14	σ	C11-C15	σ*	4.82	1.08	0.064
C21-H25	σ	C16-C18	σ*	3.99	1.09	0.059
C14-C15	σ	C14-N34	σ*	2.94	1.18	0.053

C14-C15	σ	C15-C26	σ*	1.99	1.19	0.044
C3-C26	σ	C3-C4	σ*	1.83	1.18	0.041
C12-N33	σ	C30-N33	σ*	1.33	1.38	0.038
C11-C16	σ	C12-C13	σ*	1.2	1.11	0.033
C17-C19	σ	C17-H20	σ*	1.15	1.15	0.033
N37-O39	σ	C23-N37	σ*	1	1.37	0.034
C5-C6	σ	C6-H10	σ*	0.92	1.14	0.029
C12-N33	σ	C11-C15	σ*	0.83	1.27	0.029
C23-N37	σ	C19-C23	σ*	0.79	1.36	0.029
C3-C26	σ	C15-C26	σ*	0.72	1.08	0.025
C1-H7	σ	C1-C6	σ*	0.69	1.1	0.025
C17-H20	σ	C19-H24	σ*	0.62	0.97	0.022
C26-O27	σ	C14-C15	σ*	0.55	1.67	0.027
C12-C13	σ	C11-C16	σ*	0.53	1.12	0.022
N34	LP(1)	C14-C15	π*	43.94	0.32	0.108
N34	LP(1)	C13-N32	π*	42.82	0.27	0.098
O39	LP(2)	C18-C21	σ*	0.54	0.86	0.02
N33	LP(1)	C13-N34	σ*	0.52	0.76	0.018

Table S6: NBO table of compound **7**.

**Donor(*i*)**	**Type**	**Acceptor(*j*)**	**Type**	***E*(2)^a**	***E*(J)E(i)^b**	**F(i,j)^c**
C13-N32	Π	C28-C30	π*	23.94	0.34	0.08
C16-C18	Π	C17-C19	π*	21.98	0.28	0.07
C28-C30	Π	C12-N33	π*	19.59	0.28	0.066
C2-C3	Π	C1-C6	π*	18.86	0.28	0.066
C28-C30	Π	C13-N32	π*	17.54	0.26	0.062
C21-C23	Π	C17-C19	π*	16.89	0.3	0.063
C13-N32	Π	C12-N33	π*	15.65	0.33	0.065
C2-C3	Π	C26-O27	π*	14.38	0.29	0.06

C4-C5	Π	C14-C15	π*	13.74	0.3	0.058
C26-O27	Π	C14-C15	π*	4.68	0.4	0.041
C26-O27	Π	C2-C3	π*	4.39	0.41	0.04
C14-C15	Π	C14-C15	π*	2.97	0.31	0.028
C5-C6	Σ	C4-C14	σ*	5.79	1.15	0.073
C30-H31	Σ	C12-N33	σ*	5.14	1.08	0.066
C1-C2	Σ	C3-C26	σ*	4.97	1.11	0.067
C3-C26	Σ	C4-C5	σ*	4.38	1.2	0.065
C18-C21	Σ	C16-C18	σ*	4	1.28	0.064
C21-C23	Σ	C18-C21	σ*	3.44	1.29	0.06
C14-C15	Σ	C14-N34	σ*	2.99	1.18	0.053
C2-C3	Σ	C4-C14	σ*	2.16	1.16	0.045
C14-N34	Σ	C13-N32	σ*	1.99	1.37	0.047
C28-N32	Σ	C28-C30	σ*	1.53	1.39	0.041
C11-C15	Σ	C11-H36	σ*	0.98	1.04	0.029
C14-C15	Σ	C11-C16	σ*	0.64	1.13	0.024
N34	LP(1)	C13-N32	π*	42.55	0.27	0.098
O37	LP(2)	C21-C23	π*	30.25	0.34	0.097
O27	LP(2)	C15-C26	σ*	19.48	0.72	0.107
N32	LP(1)	C12-C13	σ*	10.78	0.87	0.087
O37	LP(1)	C21-C23	σ*	7.16	1.11	0.08
N32	LP(1)	C13-N34	σ*	4.73	0.78	0.055
O37	LP(1)	C38-H40	σ*	3.04	0.94	0.048
N33	LP(1)	C13-N34	σ*	0.52	0.76	0.018

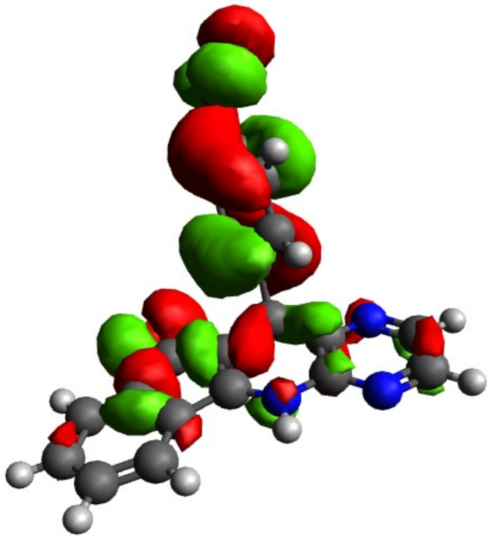

HOMO -1

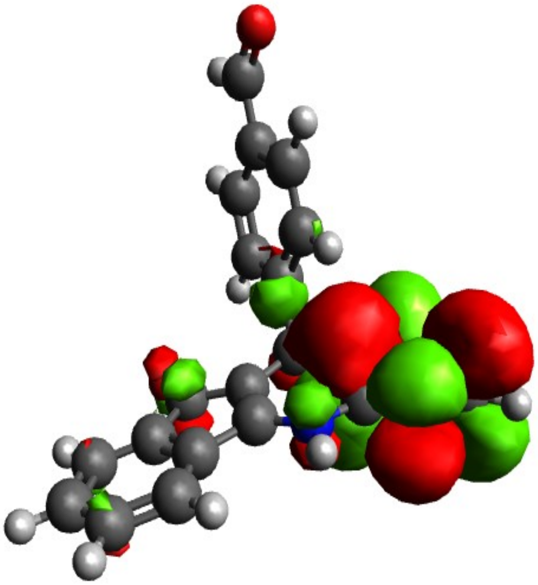

LUMO +1

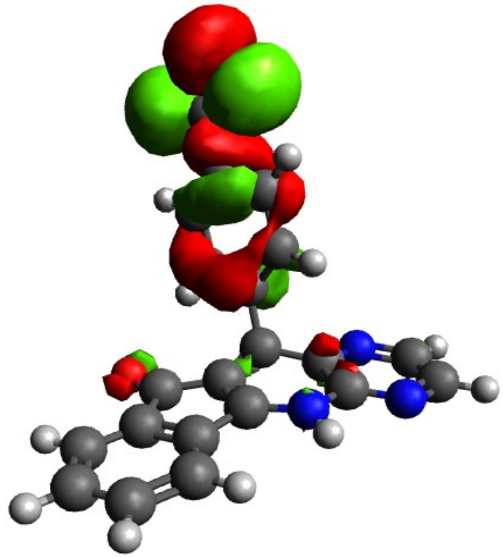

HOMO -2

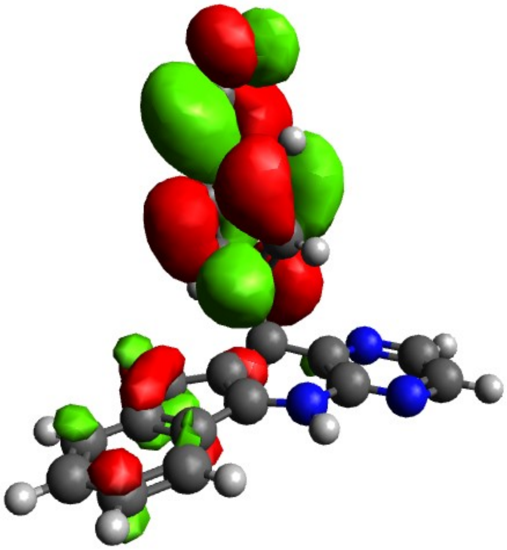

LUMO +2

D4

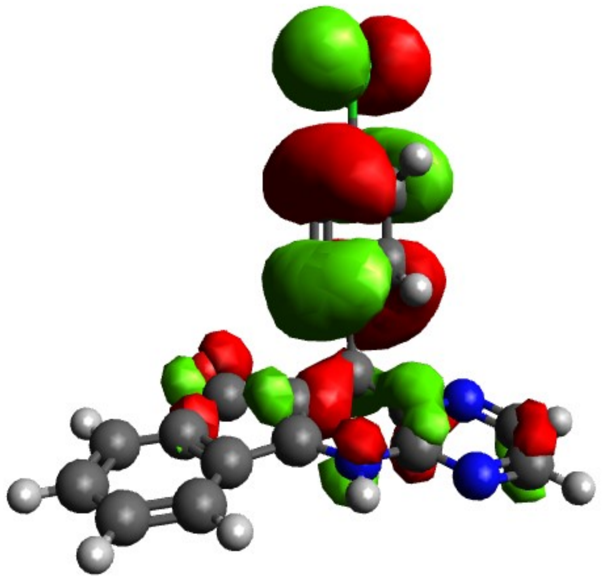

HOMO -1

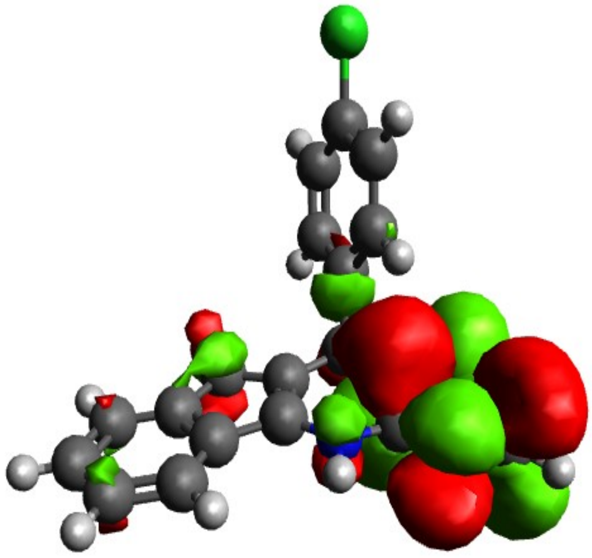

LUMO +1

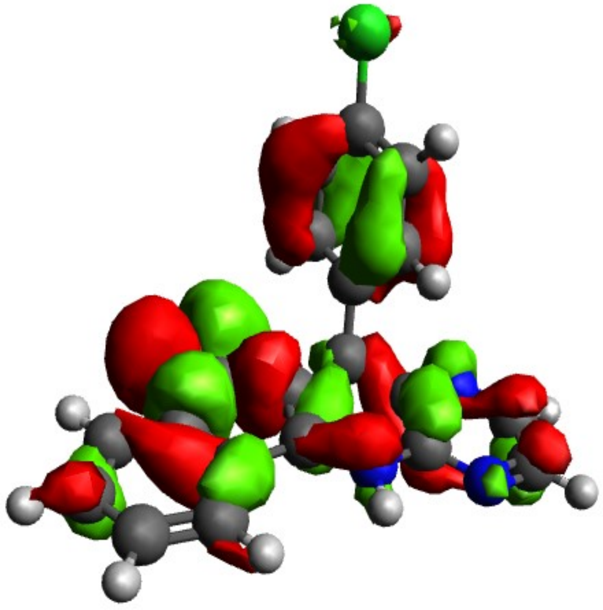

HOMO -2

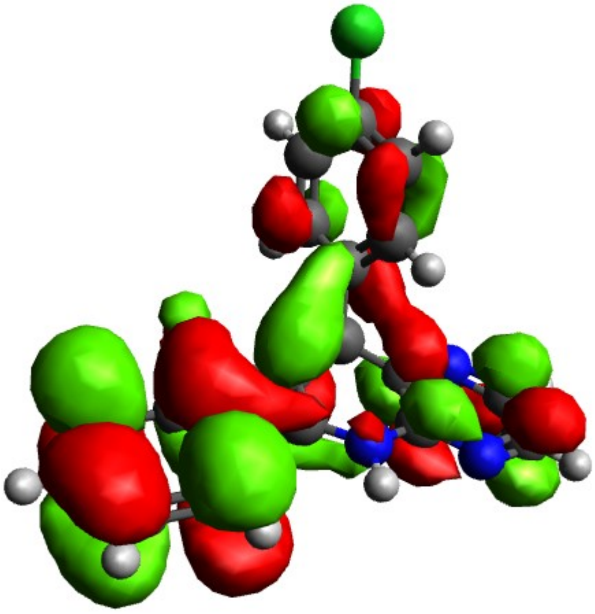

LUMO +2

D5

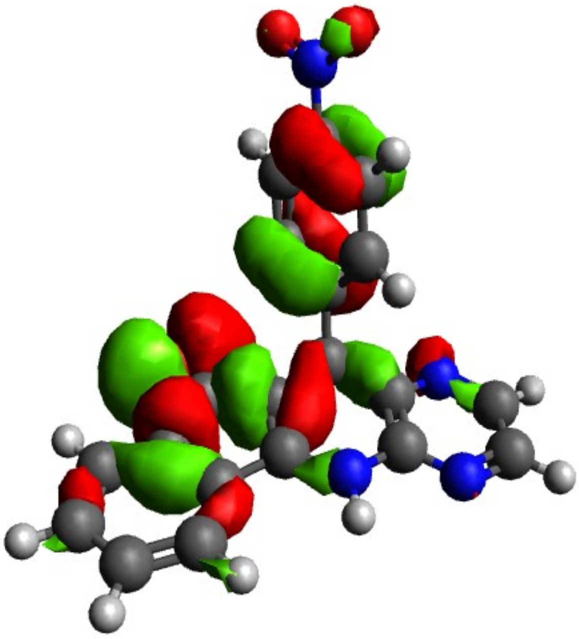

HOMO -1

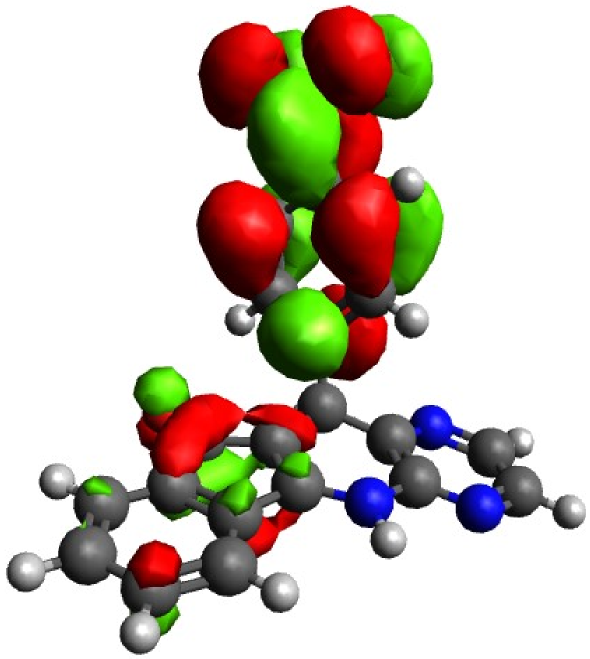

LUMO +1

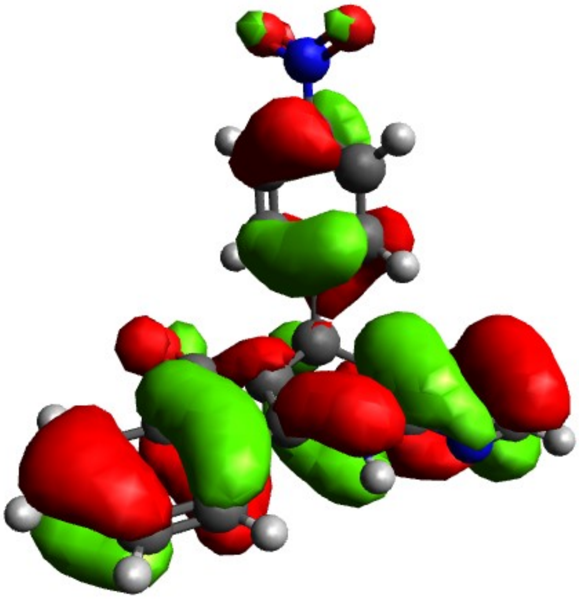

HOMO -2

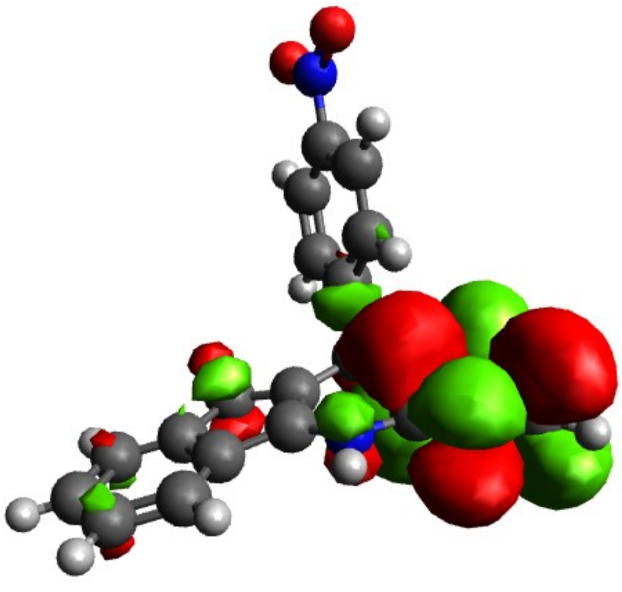

LUMO +2

D6

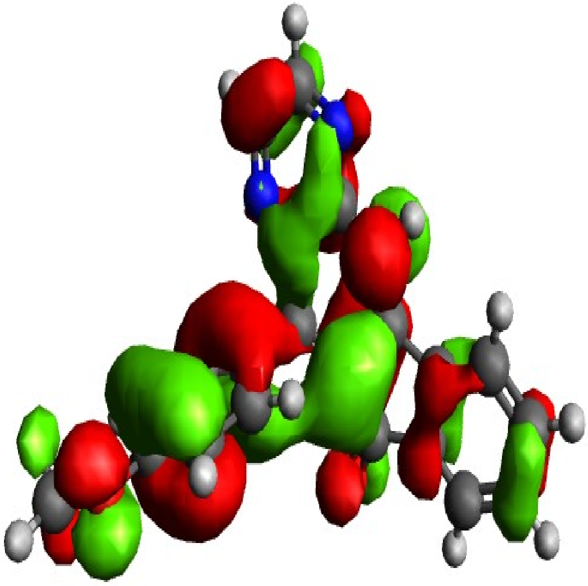

HOMO -1

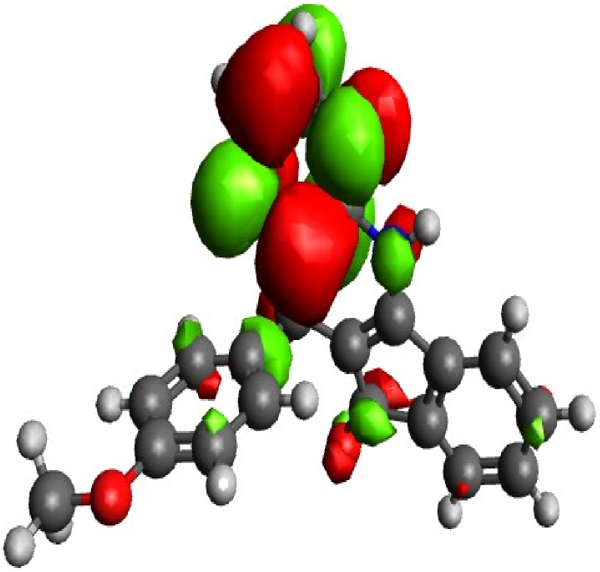

LUMO +1

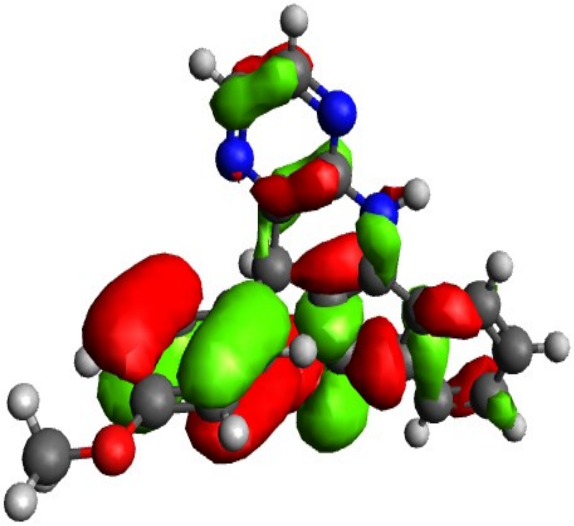

HOMO -2

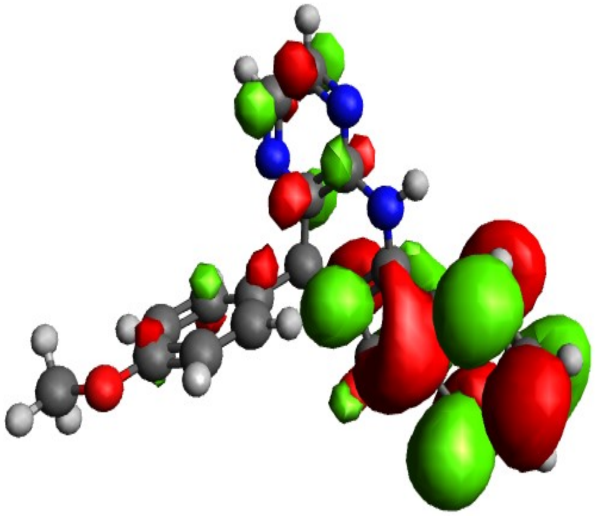

LUMO +2

D7

Figure S1: HOMO-1, LUMO+1, HOMO-2 and LUMO+2 of the studied compounds **4 to 7.**

Table S7: Computed energies (*E*) and energy gap (E_{LUMO}-E_{HOMO}) of HOMO and LUMO of investigated compounds in *eV*.

Compounds	E_{HOMO}	E_{LUMO}	E_{gap}
**4**	-6.131	-2.665	3.466
**5**	-6.068	-2.604	3.646
**6**	-6.261	-2.789	3.472
**7**	-5.858	-2.414	3.444

Table S8: Calculated energies (E) and energy gap (ΔE) of HOMO-1, LUMO+1, HOMO-2 and LUMO+2 for **D4-D7.**

**Comp.**	**HOMO-1**	**LUMO+1**	**ΔE**	**HOMO-2**	**LUMO+2**	**ΔE**
**D4**	-6.967	-1.895	5.072	-6.978	-1.765	5.213
**D5**	-6.647	-1.848	4.799	-7.052	-1.04	6.012
**D6**	-7.192	-2.377	4.815	-7.306	-2.028	5.278
**D7**	-5.965	-2.414	3.551	-6.834	0.867	5.967

Table S9: Global reactivity parameters of (**4-7)** compounds.

**Comp.**	***IP***	***EA***	***X***	***η***	***μ***	***ω***	***σ***	**ΔN_{max}**
**D4**	6.131	2.665	4.398	1.733	-4.398	5.580	0.288	2.537
**D5**	6.068	2.604	4.336	1.732	-4.336	5.427	0.288	2.503
**D6**	6.261	2.789	4.525	1.736	-4.525	5.897	0.288	2.606
**D7**	5.858	2.414	4.136	1.722	-4.136	4.967	0.290	2.401

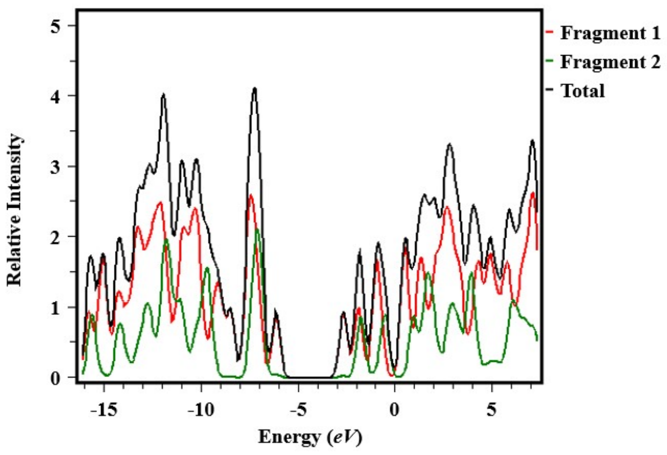

4

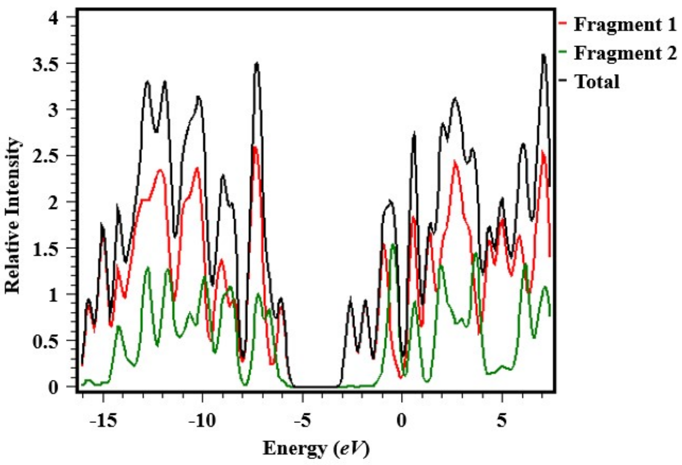

5

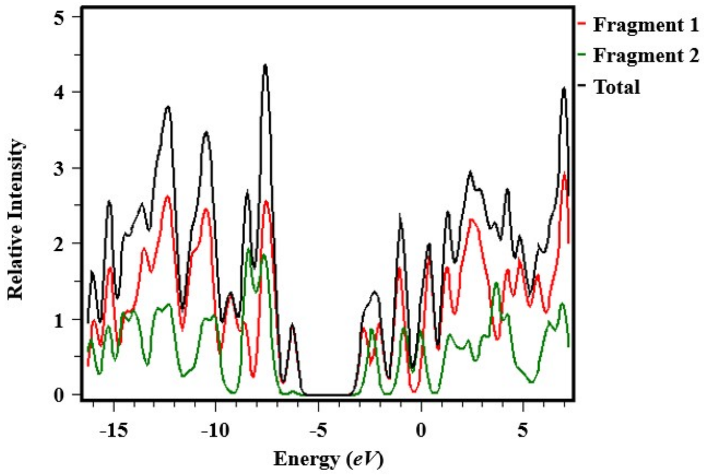

6

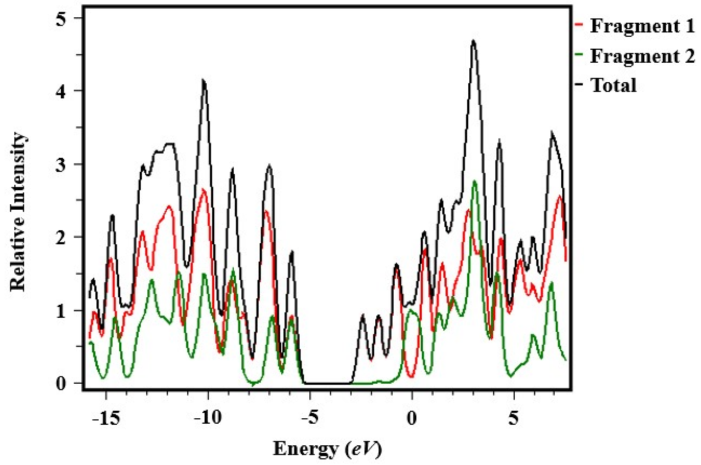

7

Figure S2: DOS graphs of **4-7** compounds.

Table S10: DOS %age of HOMO and LUMO for **4-7** compounds.

**HOMO DOS %age**		
**Compounds**	**Fragment 1**	**Fragment 2**
4	94.7	5.3
5	92.7	7.3
6	95.3	4.7
7	52.6	47.4
**LUMO DOS %age**		
**Compounds**	**Fragment 1**	**Fragment 2**
4	97.3	2.7
5	99.0	1.0
6	92.9	7.1
7	99.5	0.5

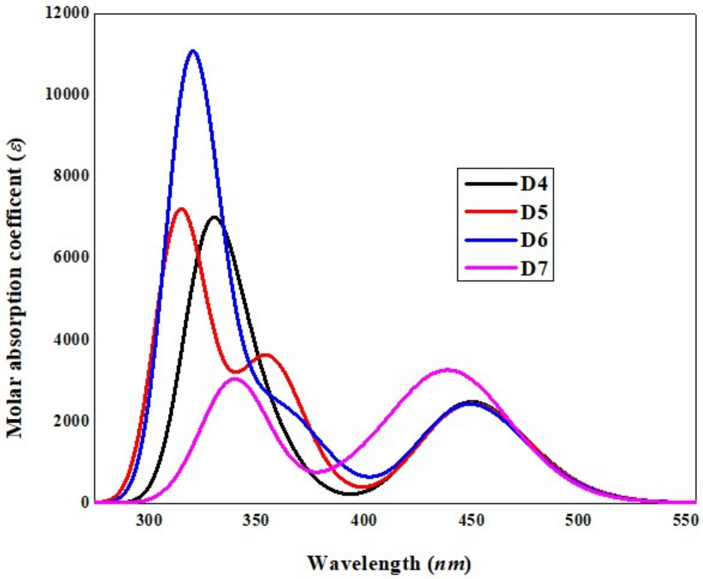

Figure S3: Absorption spectra of compounds 4-7 in gaseous phase.

Table S11: Transition energy (*E*), maximum absorption wavelength (λ_{max}), oscillator strength (f_{os}), and major transition contributions of studied compounds (**4-7**).

Comp.	DFT λ (*nm*)	E(*eV*)	*f*	MO contributions
**4**	323.693	3.830	0.083	H-3→L (26%), H-2→L (11%), H-1→L (26%), H→L+2 (29%), H-5→L (2%), H-4→L (4%)
**5**	310.131	3.998	0.092	H-4→L (23%), H-3→L (46%), H-2→L (10%), H-6→L (2%), H-5→L (8%), H-5→L+1 (2%), H→L+2 (5%)
**6**	315.417	3.931	0.152	H-2→L (90%), H-1→L (2%)
**7**	443.704	2.794	0.031	H-1→L (10%), H→L (87%),

MO=molecular orbital; H=HOMO, L=LUMO

Table S12: UV-Visible analysis of compound **4.**

NO	DFT λ (nm)	E(eV)	*f*	MO contributions
1	445.682	2.782	0.034	H→L (97%),
2	396.078	3.130	0.000	H-4→L (10%), H-3→L (33%), H-1→L (48%), H-5→L (3%)
3	348.966	3.553	0.021	H→L+1 (98%),
4	339.227	3.655	0.000	H-2→L (24%), H-2→L+2 (60%), H-2→L+8 (2%), H-1→L+2 (8%)
5	330.079	3.756	0.010	H-3→L (18%), H-1→L (11%), H→L+2 (66%),

6	323.693	3.830	0.083	H-3→L (26%), H-2→L (11%), H-1→L (26%), H→L+2 (29%), H-5→L (2%), H-4→L (4%)

Table S13: UV-Visible analysis of compound **5.**

NO	DFT λ (nm)	E(eV)	*f*	MO contributions
1	445.762	2.781	0.034	H→L (97%),
2	396.318	3.128	0.001	H-2→L (68%), H-1→L (18%), H-3→L (9%)
3	352.448	3.518	0.023	H-2→L (13%), H-1→L (60%), H→L+1 (26%),
4	349.576	3.547	0.026	H-1→L (21%), H→L+1 (73%), H-2→L (4%)
5	310.131	3.998	0.092	H-4→L (23%), H-3→L (46%), H-2→L (10%), H-6→L (2%), H-5→L (8%), H-5→L+1 (2%), H→L+2 (5%)
6	307.447	4.033	0.007	H-5→L (64%), H-5→L+1 (17%), H-3→L (13%),

Table S14: UV-Visible analysis of compound **6**.

NO	DFT λ (nm)	E(eV)	*f*	MO contributions
1	444.738	2.788	0.034	H→L (97%),
2	393.938	3.147	0.000	H-1→L (86%), H-5→L (3%), H-1→L+1 (3%)
3	368.343	3.366	0.015	H→L+1 (98%),
4	349.232	3.550	0.022	H→L+2 (98%),
5	323.685	3.830	0.000	H-7→L (10%), H-7→L+1 (85%), H-7→L+6 (3%)
6	315.417	3.931	0.152	H-2→L (90%), H-1→L (2%)

Table S15: UV-Visible analysis of compound **7**.

NO	DFT λ (nm)	E(eV)	*f*	MO contributions
1	443.704	2.794	0.031	H-1→L (10%), H→L (87%),
2	420.12	2.951	0.018	H-2→L (11%), H-1→L (75%), H-3→L (4%), H→L

	9			(8%)
3	390.858	3.172	0.008	H-3→L (23%), H-2→L (54%), H-1→L (13%), H-4→L (3%), H→L (4%)
4	347.723	3.566	0.007	H→L+1 (94%), H-1→L+1 (4%)
5	335.537	3.695	0.033	H-1→L+1 (93%), H→L+1 (4%)
6	319.390	3.882	0.008	H-3→L (69%), H-2→L (28%),

Table S16: Calculated vibrational frequencies of **4**.

ᵃFreq	*ᵃI$_{IR}$*	*EXP*	**Vibrational assignments**
3614	66		υ(N-H)
3194	20	3214	υ(s) C-H_{Ben}
3185	10		υ(s) + υ(as) C-H_{Ben}
3186	6		υ(as) C-H_{Ben}
2873	162	2894	υ (C-H-O)
1778	301		υ(C-O)
1664	71	1660	υ (C=C-C=C_{Ben}) + υ(C-O)
1585	24		υ (C=N-C=N_{pyr}) + (ρ) C-H_{pyr} + υ(C-O)
1544	327	1566	υ(C-O) + υ (C-H)
1474	86		(ρ + δ) C-H_{Ben} + (ρ) C-H_{pyr}
1435	359		υ(C-N) + (ρ) C-H_{Ben} + υ(C-C)
1357	34		(ρ) C-H_{pry} + γ (C-H_{Ben})
1335	24		(ρ) C-H_{Ben}
1233	61		(ρ + δ) C-H_{Ben}
1197	121		(ρ + δ) C-H_{Ben}
1090	14		(ρ + δ) C-H_{Ben} + (δ) C-H_{pyr}
944	13		υ (C=N-C=N_{pyr}) + γ (C-H_{pry})
892	54	843	(ρ) C-H_{Ben} + (ρ) (N-H) + γ(C-H-O)
771	38		(*w*) C-H_{Ben}
619	16		(*w*) C-H_{Ben} + (ρ) C-H_{Ben}

Table S17: Calculated vibrational frequencies of **5**.

ᵃFreq	*ᵃI$_{IR}$*	*EXP*	**Vibrational assignments**
3614	64		υ(N-H)
3193	20	3193	υ(s) C-H_{Ben}
3172	6		υ(s) + υ(as) C-H_{Ben}
3160	3		υ(as) C-H_{Ben}
1763	191		υ(C-O)
1664	69	1661	υ (C=C-C=C_{Ben}) + υ(C-O)+ υ υ (C=N-C=N_{pyr})
1632	107		υ (C=C-C=C_{Ben}) + υ(C-O)+ υ (C=N-C=N_{pyr})
1543	321		(ρ)(N-H)+ υ (C=N-C=N_{pyr})

1435	335		(δ) (C-H_{pyr}) +(ρ) C-H_{Ben} + υ(C-C)
1372	51		(δ) (C-H_{pyr}) +(ρ) C-H_{Ben}
1269	46		(δ) (C-H_{pyr}) +(ρ) C-H_{Pry}
1197	122		(ρ + δ) C-H_{Ben} + γ (C-H_{Ben})
1143	52		(ρ + δ) C-H_{Ben}
1030	41		υ (C=C-C=C_{Ben}) + (ρ) C-H_{Ben}
943	11	958	(τ + δ) C-H_{Ben}
892	51		(ρ) C-H_{Ben} + (ρ) C-H_{Pry} + (ρ) (N-H)
770	23	759	(*w*) C-H_{Ben} + υ(C-Cl)
690	23		(γ) C-H_{Ben}

Table S18: Calculated vibrational frequencies of **6**.

***a*Freq**	***a*I_{IR}**	***EXP***	**Vibrational assignments**
3613	66		υ(N-H)
3224	2	3347	υ(s) C-H_{Ben}
3180	39		υ(as) C-H_{Ben}
3174	5		υ(s) + υ(as) C-H_{Ben}
3032	7		υ(C-H_{Pry})
1762	190		υ(C-O)
1664	70	1659	υ (C=C-C=C_{Ben}) + υ(C-O)+ υ υ (C=N-C=N_{pyr})+ (δ) C-H_{Ben}
1652	68	1565	υ (C=C-C=C_{Ben}) + υ(C-O)+ υ (N-O_{NO2})
1544	313		(ρ)(N-H)+ υ (C=N-C=N_{pyr}) + (δ) C-H_{Ben}
1435	351		(δ) (C-H_{pyr}) +(ρ) C-H_{Ben} + (δ) C-H_{Ben} + + υ(C-C)
1378	389	1338	(δ) (C-H_{pyr}) +(ρ) C-H_{Ben} + υ (N-O_{NO2})
1271	66		(δ) (C-H_{pyr}) +(ρ) C-H_{Pry}
1197	118		(ρ + δ) C-H_{Ben} + γ (C-H_{Ben})
1143	54		(ρ + δ) C-H_{Ben}
1090	14		υ (C=C-C=C_{Ben}) + (ρ) C-H_{Ben} + (δ) (C-H_{pyr})
945	17	958	(τ + δ) C-H_{Ben}
892	52	895	(ρ) C-H_{Ben} + (ρ) C-H_{Pry} + (ρ) (N-H)
771	25	765	(*w*) C-H_{Ben} + υ(N-O_{NO2})
684	6		(ρ) C-H_{Pry} + (ρ) (N-O_{NO2})

Table S19: Calculated vibrational frequencies of **7**.

***a*Freq**	***a*I_{IR}**	***EXP***	**Vibrational assignments**
3615	62		υ(N-H)
3202	12	3459	υ(s) C-H_{Ben}
3192	22		υ(s) C-H_{Ben}
3183	11		υ(s) + υ(as) C-H_{Ben}
3177	46		υ(as) C-H_{pyr}
3130	28		υ(s) OCH_{CH3}

2998	65		υ(s) OCH_{CH3}
1771	207		υ(C-O)
1665	67	1658	υ (C=C-C=C_{Ben}) + υ(C-O)+ υ υ (C=N-C=N_{pyr})
1632	114		υ (C=C-C=C_{Ben}) + (ρ + δ) C-H_{Ben} + υ (C=N-C=N_{pyr})
1544	304		(ρ)(N-H)+ υ (C=N-C=N_{pyr})
1433	354		(δ) (C-H_{pyr}) +(ρ) C-H_{Ben} + + υ(C-C)
1378	58		(δ) (C-H_{pyr}) +(ρ) C-H_{Ben}
1275	128	1270	(δ) (C-H_{pyr}) +(ρ) C-H_{Pry}
1194	115	1187	(ρ + δ) C-H_{Ben} + γ (C-H_{Ben})
1140	60		(ρ + δ) C-H_{Ben}
1064	60		υ (C=C-C=C_{Ben}) + υ (OCH_{CH3})
999	4		(τ + δ) C-H_{Ben}
892	53		(ρ) C-H_{Ben} + (ρ) C-H_{Pry} + (ρ) (N-H)
772	28	720	(*w*) C-H_{Ben} + υ(OCH3)
619	16		(γ) C-H_{Ben}

Frequencies are given in cm^{-1}, υ =stretching, β=in-plane bending, γ=out-plane bending δ=scissoring, ρ=rocking, *w*= wagging, s =symmetric, as=asymmetric,τ=twisting, Ben=benzene ring, pyr = pyrazone structure, pry= prydine, EXP= Experimental values.

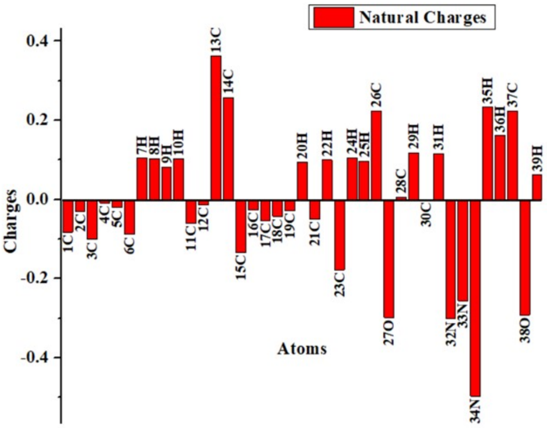

4

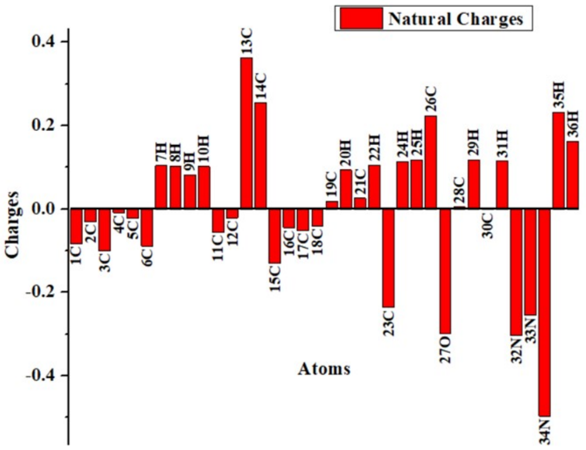

5

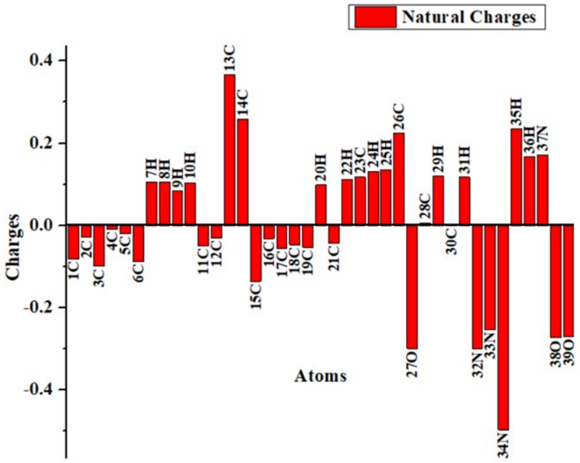

6

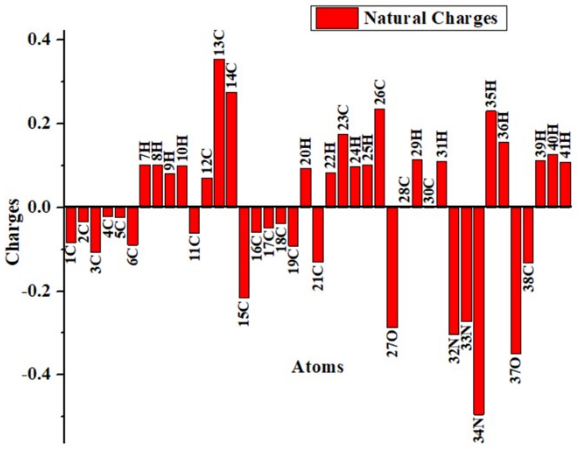

7

Figure S4: NPA graphs of **4-7** compounds.

Table 20: Computed average polarizability <α> dipole moment (μ_{tot}), first hyperpolarizability (βtot) and second hyperpolarizability (γ_{tot}) of compounds **4-7**.

**Compounds**	μ_{tot}	**<α>×10^{-23}**	β_{tot} **×10^{-30}**	**<γ>×10^{-35}**
**4**	5.9373	3.87	7.88	5.82
**5**	5.8622	3.78	9.74	5.27
**6**	8.5041	3.86	9.20	6.13
**7**	3.2695	3.90	15.6	6.63

μ_{tot} in Debye (*D*); ⟨α⟩, β_{tot}, and <γ>. are in *esu*.

Table S21: Dipole polarizability and major contributing tensor (*e.s.u.*) of the studied comp. **4-7**.

**Dipole Moment**				
	**4**	**5**	**6**	**7**
μ_x	-4.9478	-4.3304	-7.3535	-0.8698
μ_y	3.2213	3.2439	3.9722	2.3415
μ_z	-0.6282	-2.2563	-1.5704	-2.1097
μ_{total}	5.9373	5.8622	8.5041	3.2695
**Polarizability**				
α_{xx}	4.51×10^{-23}	4.40×10^{-23}	4.39×10^{-23}	4.88×10^{-23}
α_{yy}	4.29×10^{-23}	4.30×10^{-23}	4.42×10^{-23}	4.12×10^{-23}

α_{zz}	2.81×10^{-23}	2.65×10^{-23}	2.77×10^{-23}	2.72×10^{-23}
α_{total}	3.87×10^{-23}	3.78×10^{-23}	3.86×10^{-23}	3.90×10^{-23}
**2nd Hyper pol.**				
γ_X	3.67×10^{-35}	3.11×10^{-35}	3.82×10^{-35}	4.30×10^{-35}
γ_Y	1.91×10^{-35}	1.95×10^{-35}	2.09×10^{-35}	2.11×10^{-35}
γ_Z	0.23×10^{-35}	0.21×10^{-35}	0.22×10^{-35}	0.21×10^{-35}
Average<γ>	5.82×10^{-35}	5.27×10^{-35}	6.13×10^{-35}	6.63×10^{-35}
Magnitude of γ	4.15×10^{-35}	3.68×10^{-35}	4.36×10^{-35}	4.80×10^{-35}

Table S22: Frequency dependent Second hyperpolarizability (*e.s.u.*) of studied compounds **4-7**.

Parameters	Frequency ω	4	5	6	7
γ(−ω,ω,0,0)	0.000	5.82×10^{-35}	5.27×10^{-35}	6.13×10^{-35}	6.63×10^{-35}
	1907.21nm	6.19×10^{-35}	5.61×10^{-35}	6.56×10^{-35}	7.17×10^{-35}
γ(−2ω,ω,ω,0)	0.000	5.82×10^{-35}	5.27×10^{-35}	6.13×10^{-35}	6.63×10^{-35}
	1907.21nm	7.07×10^{-35}	6.43×10^{-35}	7.61×10^{-35}	8.54×10^{-35}

Table S23: The computed first hyperpolarizability (β_{tot}) and major contributing tensors (e.s.u) of compounds **4-7**.

Polarizability	4	5	6	7
β_{xxx}	-3.18×10^{-30}	4.46×10^{-30}	-7.80×10^{-30}	9.29×10^{-30}
β_{xxy}	3.19×10^{-30}	-0.59×10^{-30}	2.31×10^{-30}	-1.15×10^{-30}
β_{xyy}	2.26×10^{-30}	4.10×10^{-30}	2.00×10^{-30}	5.27×10^{-30}
β_{yyy}	4.40×10^{-30}	3.63×10^{-30}	5.07×10^{-30}	2.66×10^{-30}
β_{xxz}	0.59×10^{-30}	-0.54×10^{-30}	1.70×10^{-30}	-1.56×10^{-30}
β_{yyz}	-0.13×10^{-30}	-1.82×10^{-30}	-1.44×10^{-30}	-2.04×10^{-30}
β_{xzz}	0.15×10^{-30}	0.43×10^{-30}	0.32×10^{-30}	0.49×10^{-30}
β_{yzz}	0.18×10^{-30}	-0.13×10^{-30}	0.003×10^{-30}	0.31×10^{-30}
β_{zzz}	-0.27×10^{-31}	0.04×10^{-30}	-0.09×10^{-30}	-0.04×10^{-30}
β_{total}	7.88×10^{-30}	9.74×10^{-30}	9.20×10^{-30}	15.6×10^{-30}

Table S24: Frequency dependent First hyperpolarizability (*e.s.u.*) of studied compound **4-7**.

	Parameters	Frequency ω	4	5	6	7
**Static**	β (−ω;ω,0,)	0.000	7.88×10^{-30}	9.74×10^{-30}	9.20×10^{-30}	15.6×10^{-30}
	β (-2, ω;ω,ω)	0.000	7.88×10^{-30}	9.74×10^{-30}	9.20×10^{-30}	15.6×10^{-30}

Specific	β (−ω;ω,0)	1907.21nm	3.89×10^{-23}	3.81×10^{-23}	3.89×10^{-23}	3.93×10^{-23}
	β (−2ω;ω,ω)	1907.21nm	3.89×10^{-23}	3.81×10^{-23}	3.89×10^{-23}	3.93×10^{-23}

Table S25: *In Vitro* Antioxidant Activity of synthesized compounds **(4-7)**

**Compound**	**Conc. (µg/ml)**				**IC_{50}** (µM)
	**50**	**75**	**100**	**125**	
**4**	15.73 ± 0.84	37.81 ± 0.61	48.24 ± 1.21	68.43 ± 1.17	5.98 ± 1.95
**5**	16.92 ± 0.99	34.81 ± 1.23	63.12 ± 1.53	71.72 ± 0.88	5.92 ± 0.11
**6**	6.13 ± 0.54	22.71 ± 0.61	35.14 ± 1.21	61.43 ± 1.17	6.98 ± 0.95
**7**	6.96 ± 0.99	19.87 ± 2.02	48.86 ± 1.12	69.43 ± 2.00	7.76 ± 1.76
**Ascorbic acid**	17.34 ± 1.13	38.60 ± 0.12	69.15 ± 1.17	74.34 ± 1.08	5.31 ± 1.22

Table S26: Urease Inhibitory Activity of synthesized compounds **(4-7)**

**Compounds**	**IC_{50} (µM)**
**4**	6.79 ± 0.92
**5**	4.12 ± 1.18
**6**	4.77 ± 0.92
**7**	11.91 ± 0.34
**Thiourea**	1.94 ±0.36

Characterization By NMR & FTIR

4-(10-oxo-10,11-dihydro-5H-indeno[2',1':5,6]pyrido[2,3-b]pyrazin-11-yl)benzaldehyde (4).

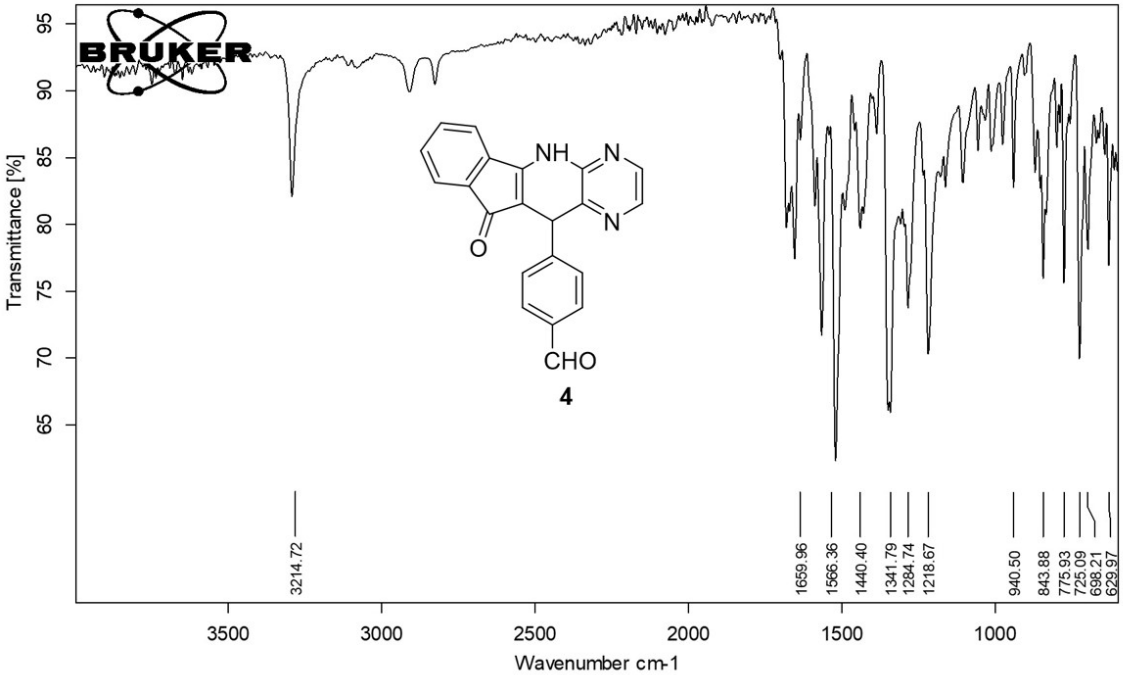

BRUKER
Transmittance [%]
95
90
85
80
75
70
65
NH
N
N
O
CHO
4
3214.72
1659.96
1566.36
1440.40
1341.79
1284.74
1218.67
940.50
843.88
775.93
725.09
698.21
629.97
3500
3000
2500
2000
1500
1000
Wavenumber cm-1

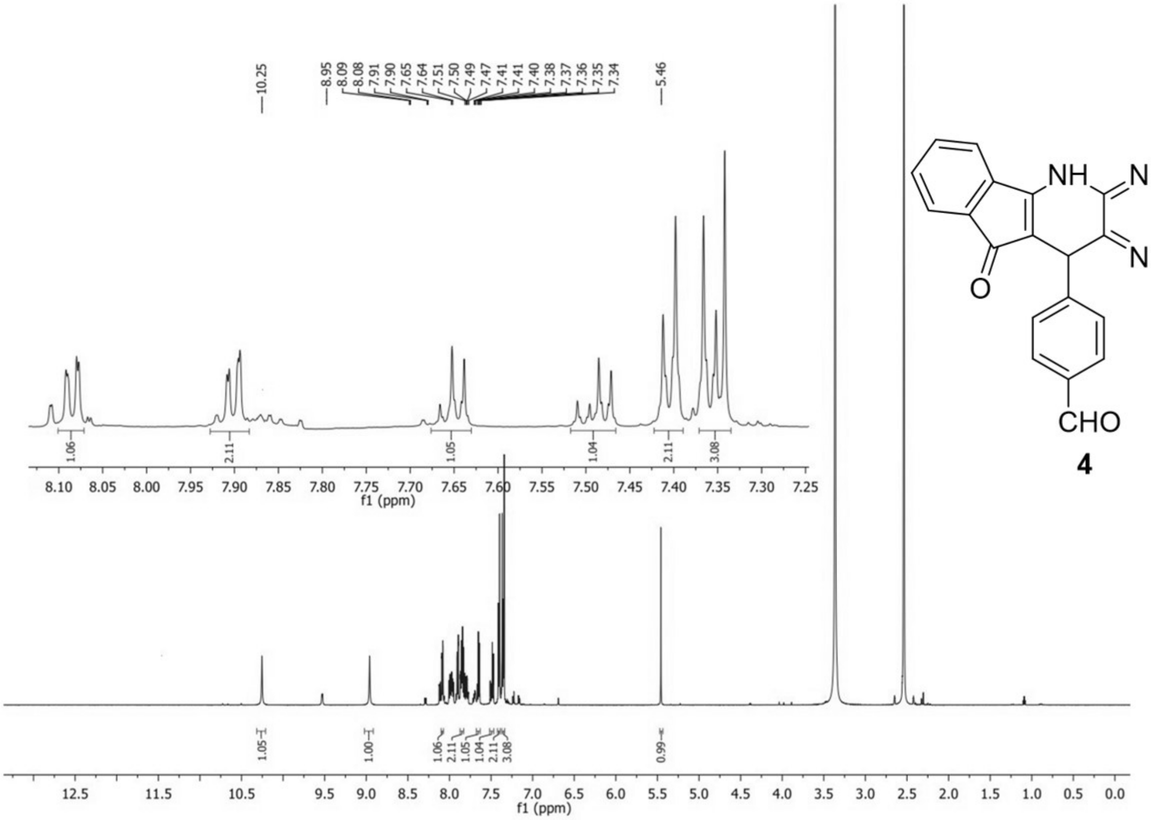

10.25
8.95
8.09
8.08
7.91
7.90
7.65
7.64
7.51
7.50
7.49
7.47
7.41
7.41
7.40
7.38
7.37
7.36
7.35
7.34
5.46
1.06
2.11
1.05
1.04
2.11
3.08
f1 (ppm)
NH
N
N
O
CHO
4
1.05
1.00
1.06
2.11
1.05
1.04
2.11
3.08
0.99
f1 (ppm)

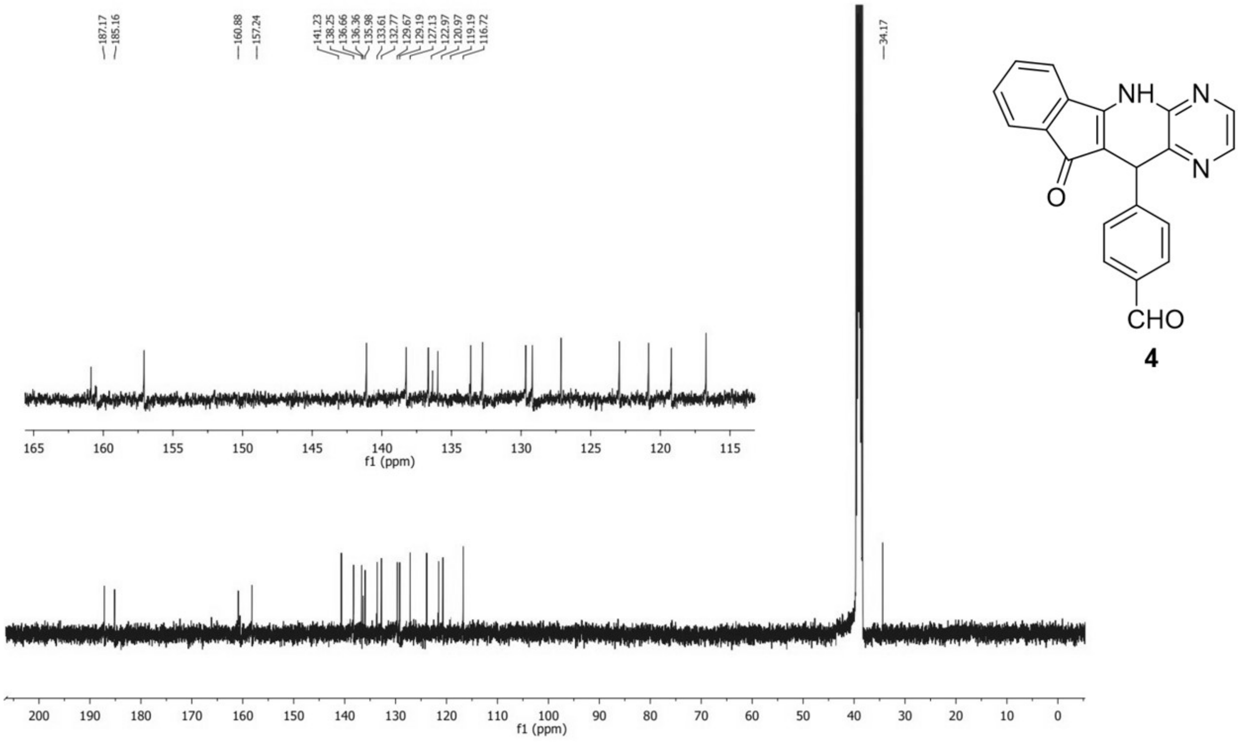

11-(4-chlorophenyl)-5,11-dihydro-10H-indeno[2',1':5,6]pyrido[2,3-b]pyrazin-10-one(5).

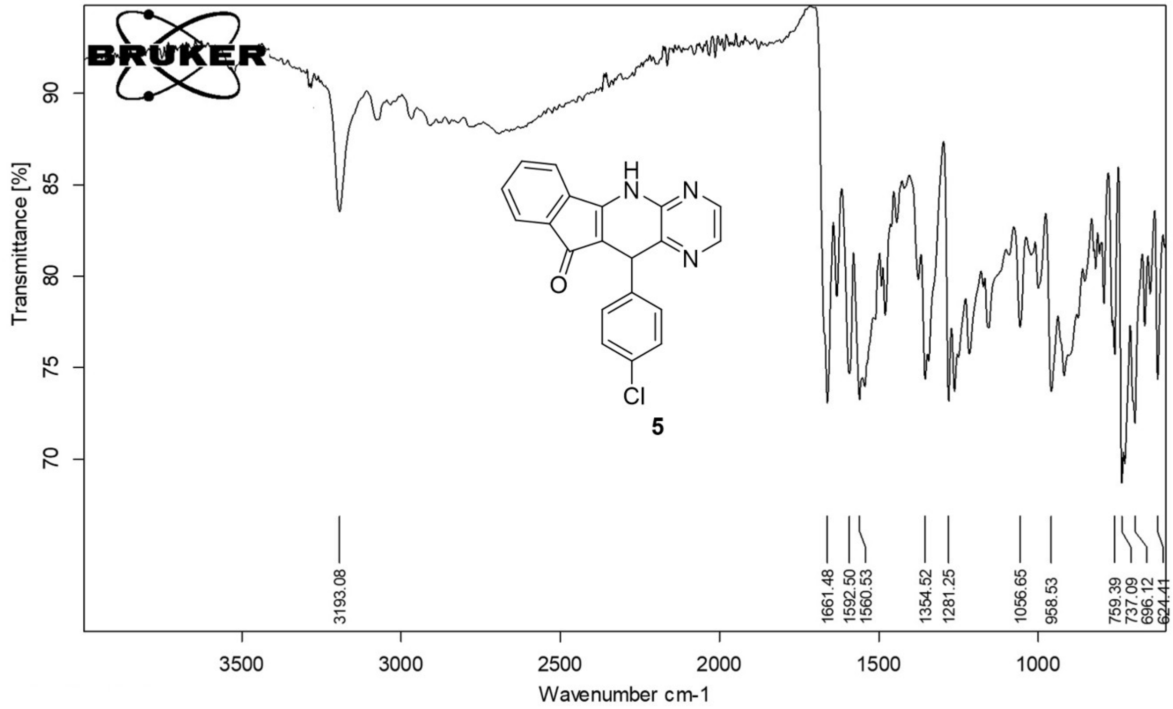

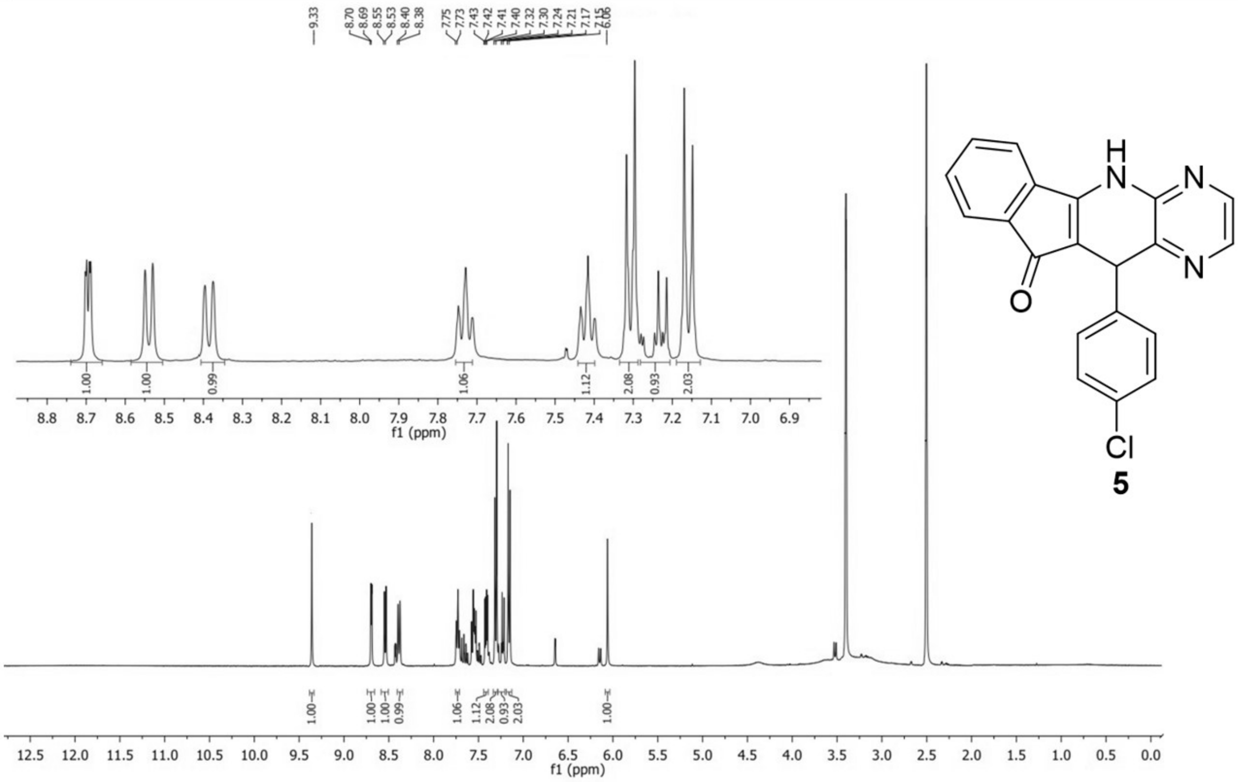
9.33
8.70
8.69
8.55
8.53
8.40
8.38
7.75
7.73
7.43
7.42
7.41
7.40
7.32
7.30
7.24
7.21
7.17
7.15
6.06
f1 (ppm)
H
N
N
O
Cl

5

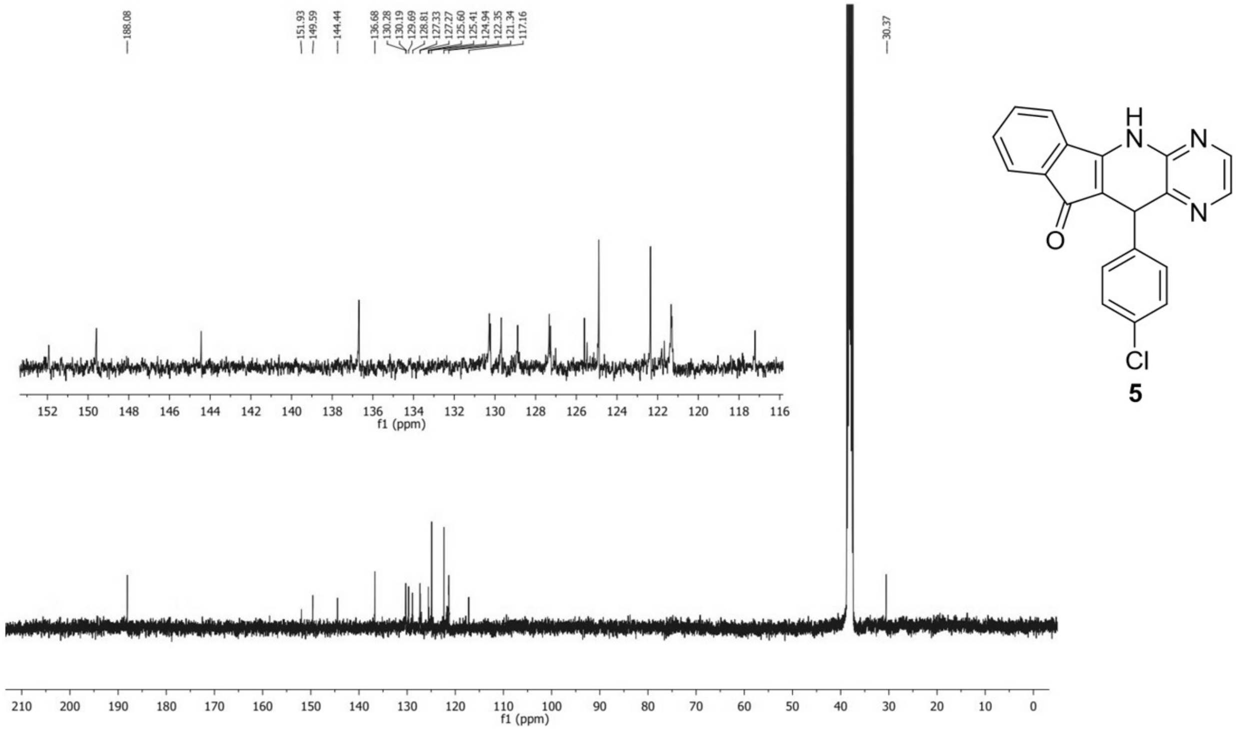
188.08
151.93
149.59
144.44
136.68
130.28
130.19
129.69
128.81
127.33
127.27
125.60
125.41
124.94
122.35
121.34
117.16
30.37
f1 (ppm)
H
N
N
O
Cl

5

11-(4-nitrophenyl)-5,11-dihydro-10H-indeno[2',1':5,6]pyrido[2,3-b]pyrazin-10-one(6).

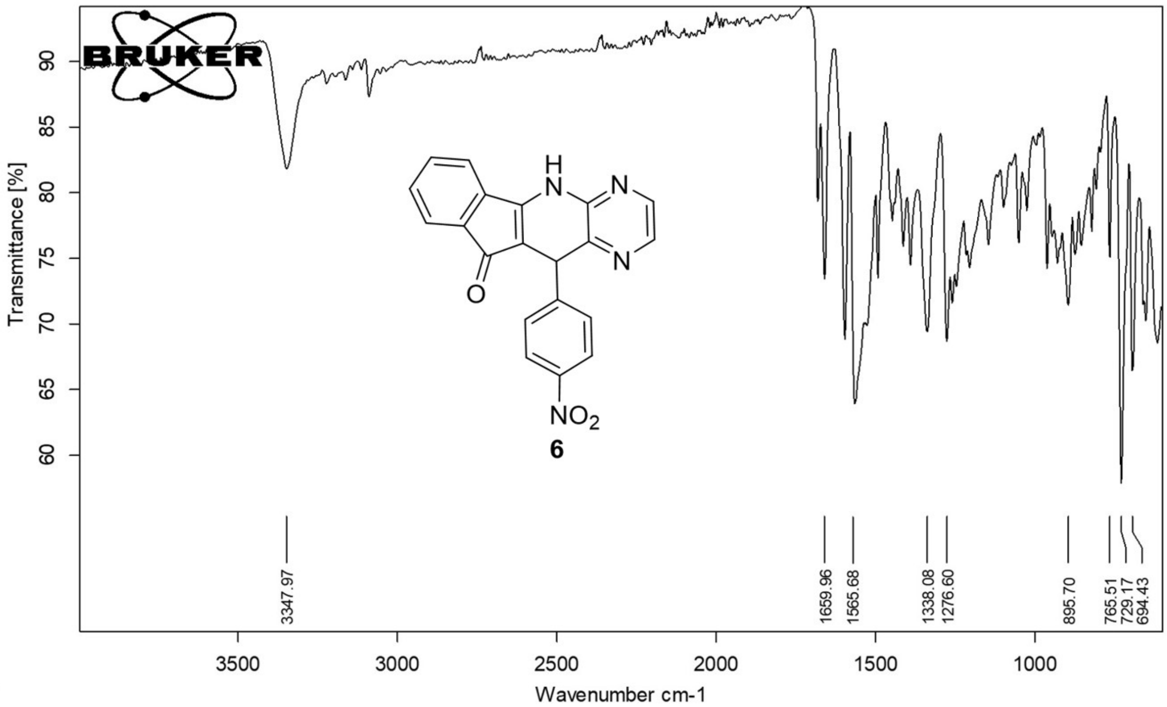

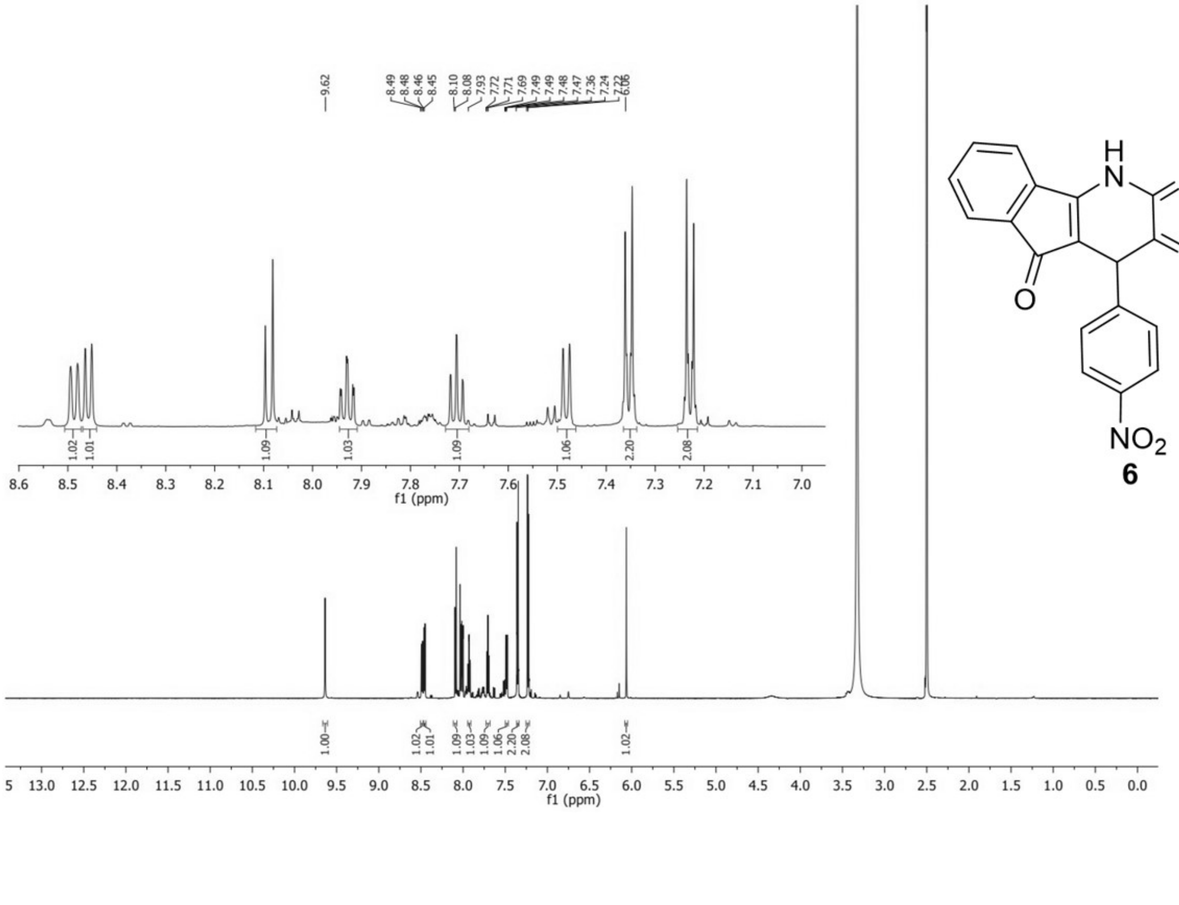

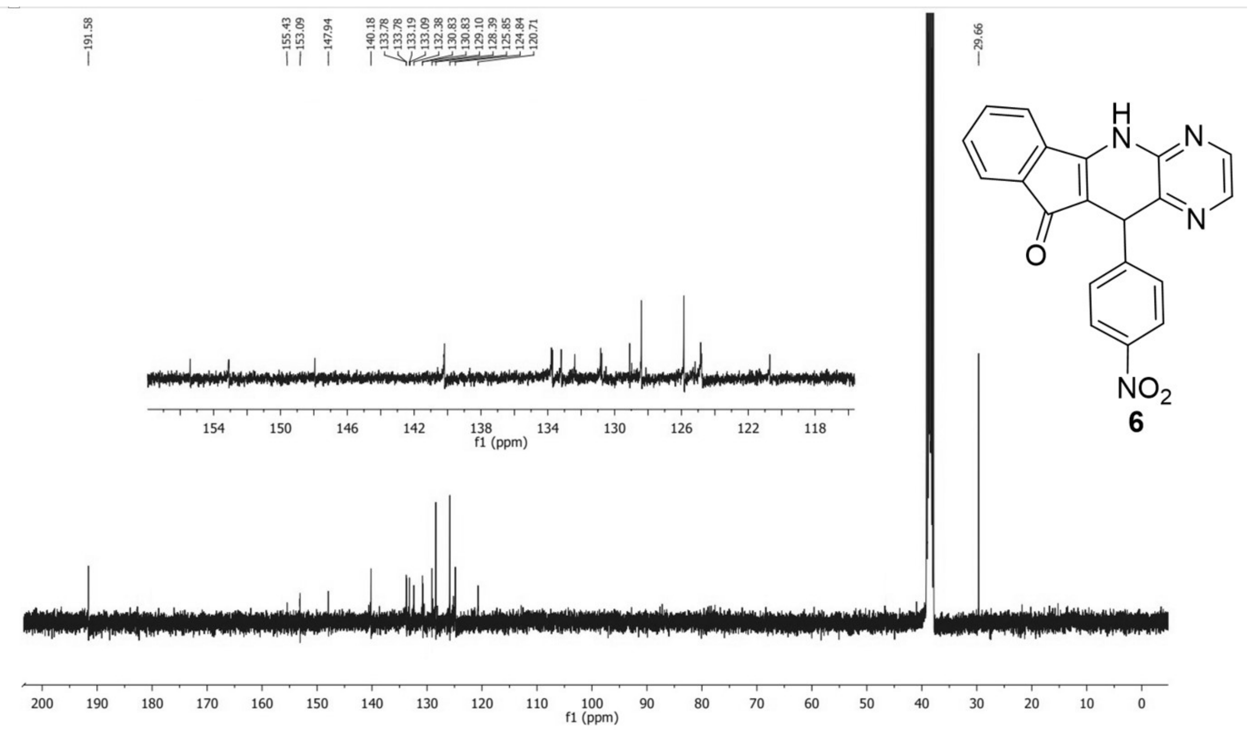

191.58
155.43
153.09
147.94
140.18
133.78
133.78
133.19
133.09
132.38
130.83
130.83
129.10
128.39
125.85
124.84
120.71
29.66
154 150 146 142 138 134 130 126 122 118
f1 (ppm)
200 190 180 170 160 150 140 130 120 110 100 90 80 70 60 50 40 30 20 10 0
f1 (ppm)
H
N
N
N
O
NO_2
6

11-(4-methoxyphenyl)-5,11-dihydro-10H-indeno[2',1':5,6]pyrido[2,3-b]pyrazin-10-one(7).

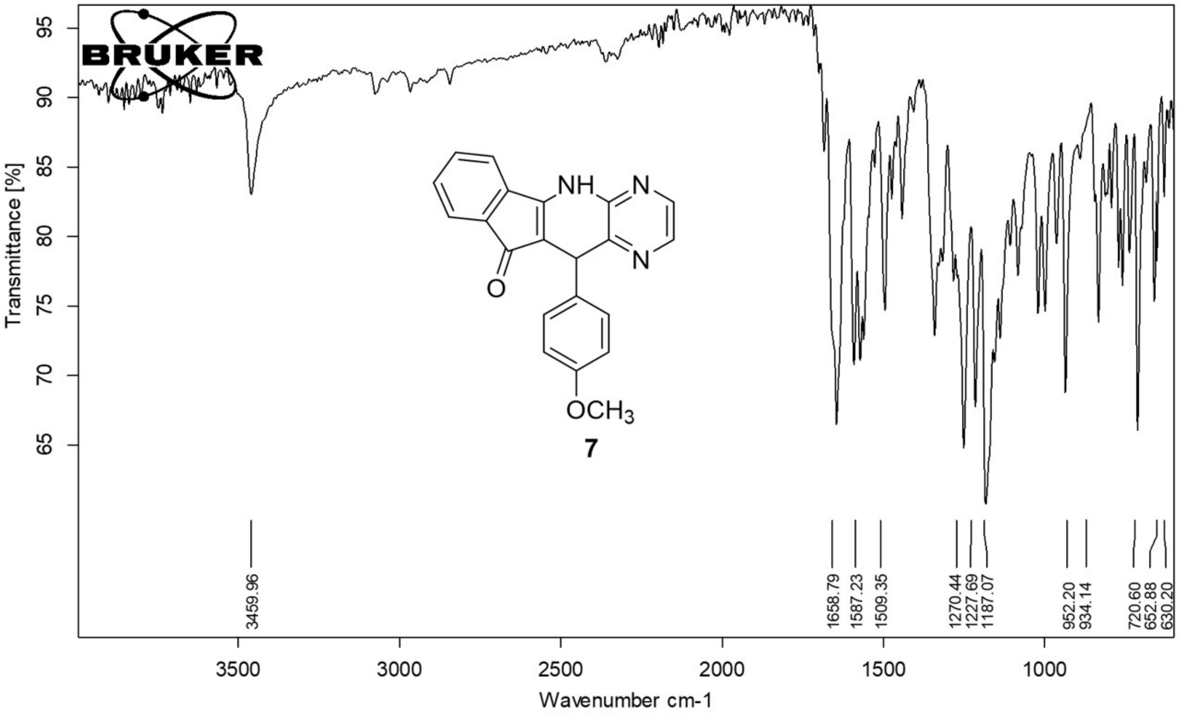

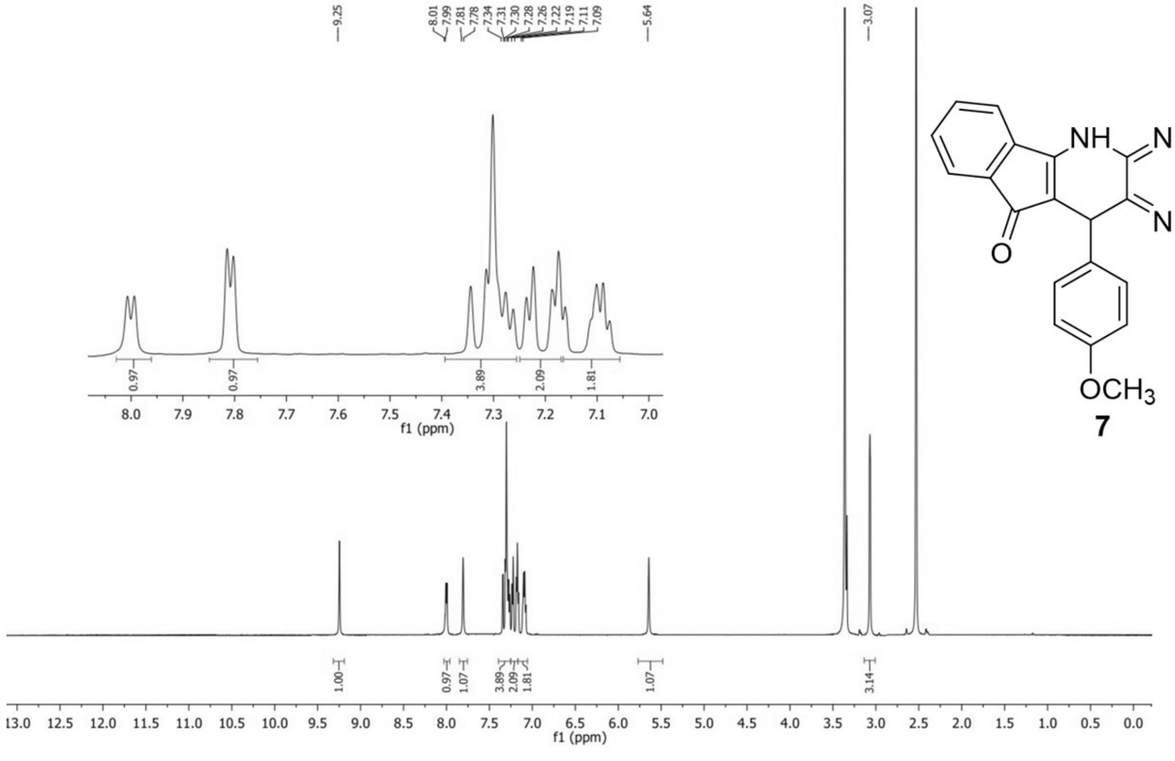

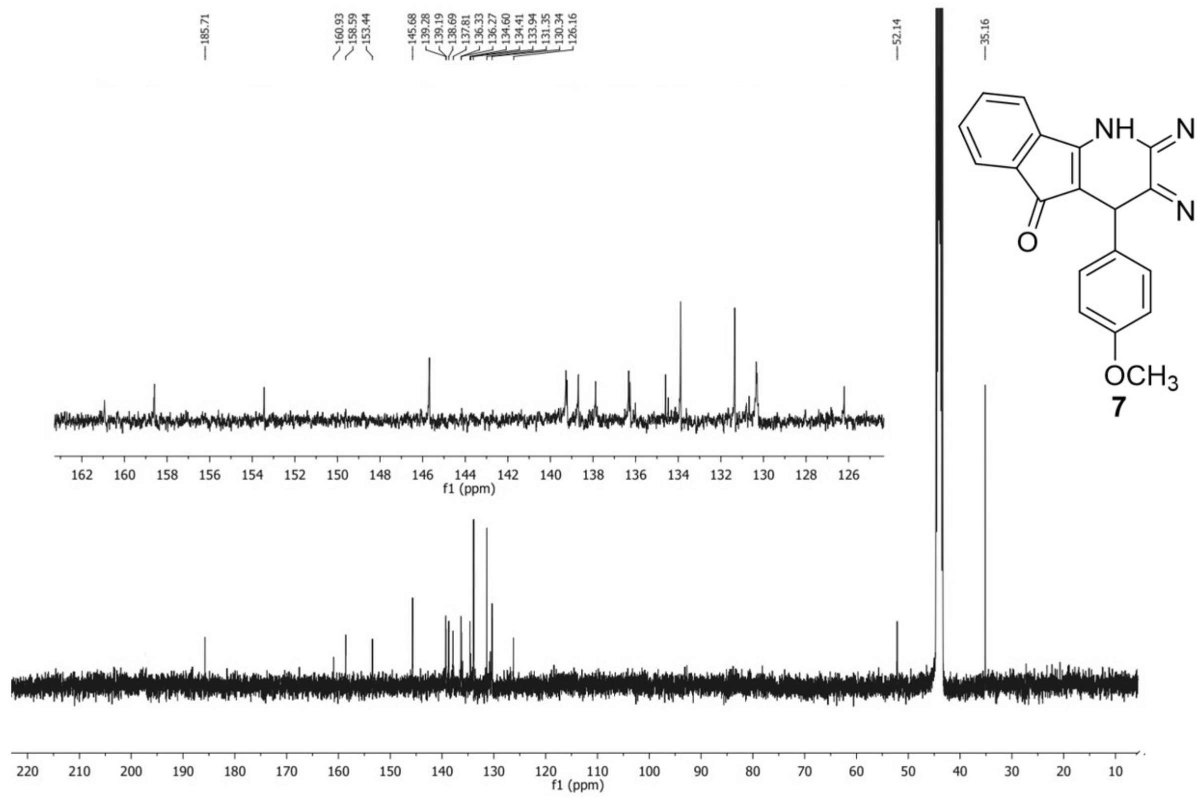

2.5.2 Characterization

4-(10-oxo-10,11-dihydro-5H-indeno[2',1':5,6]pyrido[2,3-b]pyrazin-11-yl)benzaldehyde (4).

Yellow solid, yield: 82%, mp 180°C; IR (KBr, cm^{-1}): 3214 (N-H), 2894, 2812(CHO-H), 1659.96(CHO), 1566.36(CO), 843.88, 725.09; ^{1}H-NMR (DMSO-d_6, 400 MHz): δ = 5.46 (s, 1H, Non-Aromatic-H), 7.37-7.34 (m, 3H, Indanone $C_{20,21,22}$-H), 7.40 (d, 2H, Aryl $C_{12,16}$-H, J=6.4 Hz), 7.51-7.47 (m, 1H, Indanone C_{23}-H), 7.65 (d, 1H, Pyrazine C_4-H, J=8.1 Hz), 7.90 (d, 2H, Aryl $C_{13,15}$-H, J=6.4 Hz), 8.08 (d, 1H, Pyrazine C_5-H, J=8.1 Hz), 10.25 (s, 1H, -CHO), 8.95 (s, N-H); ^{13}C-NMR (DMSO-d_6, 100 MHz): δ = 34.17 (Nonaromatic-C), 116.72, 119.19, 120.97, 122.97, 129.19, 127.13, 129.67, 132.77, 133.61, 135.98, 136.36, 136.66, 138.25, 141.23, 157.24, 160.88, 185.16 (C=O), 187.17 (CHO); Elemental analysis calculated for $C_{21}H_{13}N_3O_2$ (%): C, (74.33); H, (3.86); N, (12.38); observed (%)**:** C, (74.29); H, (3.83); N, (12.35).

11-(4-chlorophenyl)-5,11-dihydro-10H-indeno[2',1':5,6]pyrido[2,3-b]pyrazin-10-one(5).

Yellowish green solid, yield: 82%, mp 168°C; IR (KBr, cm^{-1}): 3193.08 (N-H), 1661.48 (CO), 958.53, 759.39 (C-Cl); ^{1}H-NMR (DMSO-d_6, 400 MHz): δ = 6.06 (s, 1H, Non-Aromatic-H), 7.16 (d, 2H, Aryl $C_{13,15}$-H, J=6.4 Hz), 7.23 (d, 1H, Indanone C_{20}-H), 7.31 (d, 2H, Aryl $C_{12,16}$-H, J=6.4

Hz), 7.42 (t, 1H, Indanone C_{21}-H), 7.73 (t, 1H, Indanone C_{22}-H), 8.39 (d, 1H, Indanone C_{23}-H), 8.54 (d, 1H, Pyrazine C_4-H, J=8.1 Hz), 8.70 (d, 1H, Pyrazine C_5-H, J=8.1 Hz), 9.33 (s, N-H); ^{13}C-NMR (DMSO-d_6, 100 MHz): δ = 30.37 (Nonaromatic-C), 117.16, 121.34, 122.35, 124.94, 125.41, 125.60, 127.27, 127.33, 128.81, 129.69, 130.19, 130.28, 130.68, 144.44, 149.59, 151.93, 188.08 (C=O); Elemental analysis calculated for $C_{21}H_{13}N_3O_2$ (%): C, (69.47); H, (3.50); N, (12.15); observed (%)**:** C, (69.36); H, (3.46); N, (12.11).

11-(4-nitrophenyl)-5,11-dihydro-10H-indeno[2',1':5,6]pyrido[2,3-b]pyrazin-10-one(6). Dirty green solid, yield: 85%, mp 185°C; IR (KBr, cm^{-1}): 3347.97 (N-H), 1659.96(CO), 1565.68(NO_2), 1338.08(NO_2), 895.75, 765.51; ^{1}H-NMR (DMSO-d_6, 400 MHz): δ = 6.06 (s, 1H, Non-Aromatic-H), 7.23 (d, 2H, Aryl $C_{12,16}$-H, J=6.4 Hz), 7.35 (d, 2H, Aryl $C_{13,15}$-H, J=6.4 Hz), 7.47 (d, 1H, Indanone C_{20}-H), 7.71 (t, 1H, Indanone C_{21}-H), 7.93 (t, 1H, Indanone C_{22}-H), 8.09 (d, 1H, Indanone C_{23}-H), 8.46 (d, 1H, Pyrazine C_4-H, J=8.1 Hz), 8.48 (d, 1H, Pyrazine C_5-H, J=8.1 Hz), 9.62 (s, N-H); ^{13}C-NMR (DMSO-d_6, 100 MHz): δ = 29.66 (Nonaromatic-C), 120.71, 124.84, 125.85, 128.39, 129.10, 130.82, 130.83, 132.38, 133.09, 133.19, 133.78, 133.79, 140.18, 147.94, 153.09, 155.43, 191.58 (C=O); Elemental analysis calculated for $C_{21}H_{13}N_3O_2$ (%): C, (67.41); H, (3.39); N, (15.72); observed (%)**:** C, (67.37); H, (3.34); N, (15.68).

11-(4-methoxyphenyl)-5,11-dihydro-10H-indeno[2',1':5,6]pyrido[2,3-b]pyrazin-10-one(7). Purple solid, yield: 89%, mp 178°C; IR (KBr, cm^{-1}): 3459.96 (N-H), 1658.79 (CO), 1270.44 (O-CH_3), 1187.07 (O-CH_3), 720.60; ^{1}H-NMR (DMSO-d_6, 400 MHz): δ = 5.64 (s, 1H, Non-Aromatic-H), 3.07 (s, 3H, -OCH_3), 7.10 (d, 2H, Aryl $C_{13,15}$-H, J=6.4 Hz), 7.21 (d, 2H, Aryl $C_{12,16}$-H, J=6.4 Hz), 7.32-7.29 (m, 4H, Indanone $C_{20\text{-}23}$-H), 7.79 (d, 1H, Pyrazine C_4-H, J=8.1 Hz), 8.0 (d, 1H, Pyrazine C_5-H, J=8.1 Hz), 9.25 (s, N-H); ^{13}C-NMR (DMSO-d_6, 100 MHz): δ = 35.16 (Nonaromatic-C), 52.14 (O-CH_3), 126.16, 130.34, 131.35, 133.94, 134.41, 134.60, 136.27, 136.33, 137.81, 138.69, 139.19, 139.28, 145.68, 153.44, 158.59, 160.93, 185.71 (C=O); Elemental analysis calculated for $C_{21}H_{13}N_3O_2$ (%): C, (73.89); H, (4.43); N, (12.31); observed (%)**:** C, (73.80); H, (4.37); N, (12.26).
